# Sex, tissue, and mitochondrial interactions modify the transcriptional response to rapamycin in Drosophila

**DOI:** 10.1186/s12864-024-10647-x

**Published:** 2024-08-07

**Authors:** Yevgeniy Raynes, John C. Santiago, Faye A. Lemieux, Leah Darwin, David M. Rand

**Affiliations:** 1 Department of Ecology, Evolution, and Organismal Biology, Brown University, Providence, RI 02912 USA; 2 Center for Computational Molecular Biology, Brown University, Providence, RI 02912 USA; 3 Department of Molecular Biology, Cellular Biology and Biochemistry, Brown University, Providence, RI 02912 USA

**Keywords:** mTOR, Rapamycin, Mitonuclear genotype, Tissue, Sex, Interactions, Personalized medicine

## Abstract

**Background:**

Many common diseases exhibit uncontrolled mTOR signaling, prompting considerable interest in the therapeutic potential of mTOR inhibitors, such as rapamycin, to treat a range of conditions, including cancer, aging-related pathologies, and neurological disorders. Despite encouraging preclinical results, the success of mTOR interventions in the clinic has been limited by off-target side effects and dose-limiting toxicities. Improving clinical efficacy and mitigating side effects require a better understanding of the influence of key clinical factors, such as sex, tissue, and genomic background, on the outcomes of mTOR-targeting therapies.

**Results:**

We assayed gene expression with and without rapamycin exposure across three distinct body parts (head, thorax, abdomen) of *D. melanogaster* flies, bearing either their native *melanogaster* mitochondrial genome or the mitochondrial genome from a related species, *D. simulans*. The fully factorial RNA-seq study design revealed a large number of genes that responded to the rapamycin treatment in a sex-dependent and tissue-dependent manner, and relatively few genes with the transcriptional response to rapamycin affected by the mitochondrial background. Reanalysis of an earlier study confirmed that mitochondria can have a temporal influence on rapamycin response.

**Conclusions:**

We found significant and wide-ranging effects of sex and body part, alongside a subtle, potentially time-dependent, influence of mitochondria on the transcriptional response to rapamycin. Our findings suggest a number of pathways that could be crucial for predicting potential side effects of mTOR inhibition in a particular sex or tissue. Further studies of the temporal response to rapamycin are necessary to elucidate the effects of the mitochondrial background on mTOR and its inhibition.

**Supplementary Information:**

The online version contains supplementary material available at 10.1186/s12864-024-10647-x.

## Introduction

Rapamycin, a potent macrolide derived from the bacterium *Streptomyces hygroscopicus*, was discovered on Rapa Nui in the 1960s. Initially known for its antifungal effects, rapamycin was later shown to exhibit antiproliferative and immunosuppressive properties in mammalian cells as well [1], prompting significant interest in the molecular mechanisms underlying its effects and its potential practical applications. In the ensuing decades, rapamycin has been shown to act in tandem with FKBP12 to bind to and allosterically inhibit the mechanistic target of rapamycin (mTOR) [2, 3] – a highly conserved serine/threonine kinase in the PI3K-related family [2, 4], which forms the catalytic subunit of three distinct complexes: mTORC1, mTORC2, and the recently described mTORC3 [2, 5]. mTORC1 responds to nutrient availability, hormones, growth factors, and stress to shift metabolism away from catabolic processes, such as autophagy, toward anabolism, stimulating protein and lipid synthesis, ribosome biogenesis, and cell cycle progression [2, 3]. mTORC2 is less well characterized but has been shown to regulate cell survival and cytoskeletal dynamics by activating several kinases of the AGC family and may play a role in glucose and lipid metabolism [2, 6, 7]. Rapamycin is an acute inhibitor of mTORC1 [2], blocking substrates from the mTOR catalytic site [8], and can inhibit mTORC2 following prolonged exposure to higher doses, by limiting the availability of free mTOR for incorporation into mTORC2 complexes [9, 10].

Deregulation of mTOR signaling has been implicated in a range of medical conditions, including cancer, aging, neurodegenerative diseases, and muscle-wasting disorders. To date, though, the clinical success of mTOR inhibitors like rapamycin and its analogs (rapalogs) has been relatively modest. In cancer, upregulation of mTOR signaling may stimulate tumor growth and metastasis [11]; indeed, mutations in the mTOR signaling pathway have been documented in various cancers [12, 13]. Rapalog therapy, however, has not proven very effective due in part to the inability of rapalogs to completely prevent the phosphorylation of all mTORC1 targets and the compensatory activation of alternative signaling pathways and feedback loops [1, 2, 14, 15]. Downregulation of mTOR signaling with rapamycin has also been shown to promote longevity and increase lifespan in several model systems [16–21], suggesting that mTOR inhibitors may be used to slow aging in humans as well [22]. Yet, rapamycin therapy in humans has been hindered by the adverse side effects of prolonged use, including immunosuppression, hyperlipidemia, and hyperglycemia [19, 23]. And while mTOR inhibition may aid in ameliorating neurodegenerative and muscle-wasting diseases by enhancing autophagy [24–27], the benefits of rapalog treatment are likewise countered by concerns over adverse side effects, given mTOR’s importance for muscle growth [28] and proper nervous system function [29].

Thus, despite the considerable promise of mTOR inhibitors in addressing a number of serious conditions, the multifaceted nature of mTOR signaling has constrained the development of effective therapies. Treatments aimed at complete catalytic inhibition of mTOR have proven unviable due to significant side effects, dose-limiting toxicities, and nonspecific inhibition. Instead, there is a pressing need for more targeted therapies tailored to individual patient profiles, specific tissues, and mTOR complexes [2]. Developing such personalized therapies requires a nuanced understanding of the context-dependent effects of mTOR-targeting interventions across different clinical scenarios. In other words, effective mTOR interventions must account for the influence of other clinically relevant factors on the response to treatment – for example, the possibility of one sex responding differently to a rapalog therapy than the other, or of unintended side effects manifesting in a different tissue from the one being targeted for treatment. Simply put, if rapamycin were used to treat a liver condition, what would be the effects on nerve or muscle tissue?

Here, we use *Drosophila melanogaster* to explore the influence of sex, tissue type, and retrograde signaling from mitochondria on the cellular response to rapamycin. In essence, we seek to elucidate how these variables modulate the response to mTOR inhibition, potentially leading to unintended side effects that could reduce the efficacy of mTOR antagonist therapies. The fact that sex and tissue can affect the response to rapamycin has been established in several earlier studies [17, 30–34]. Understanding the mechanistic details of these effects remains of clear clinical importance. The hypothesis that rapamycin’s effect could be modified by mitochondrial signaling is motivated by the established role of mTOR in coordinating mitochondrial activity [35–37]. Prior studies in *D. melanogaster* have demonstrated that disrupting mitonuclear communication by replacing “native” mitochondrial genomes (mtDNAs) of *D. melanogaster* with “foreign” mtDNAs from other species can alter the impact of rapamycin on mitochondrial respiration and cellular metabolite profiles [38, 39], as well as on the temporal patterns of gene expression [40].

To test whether the response to rapamycin is influenced by sex, tissue, and mitochondrial genotype, we assay *D. melanogaster* gene expression in a fully-factorial experimental design across the four key experimental factors: treatment (rapamycin vs. control), sex (male vs. female), body part (head, thorax, and abdomen), and mitonuclear communication (native vs. ‘foreign’ mtDNAs). Our experimental approach allows us to efficiently quantify the main effect of rapamycin on the expression of thousands of genes, as well as test the specific hypothesis that interactions between rapamycin treatment and each of the other factors (sex, tissue, and mtDNA) have distinct influence on the transcriptional response to rapamycin. Our findings broadly confirm the expected first-order effects of rapamycin. We also uncover significant influence of sex and tissue on the transcriptomic effect of rapamycin (i.e., sex-by-treatment and tissue-by-treatment interactions), with subtle signs of interaction between rapamycin treatment and mitochondrial genotype in our experimental system.

## Results

**Fig. 1:**
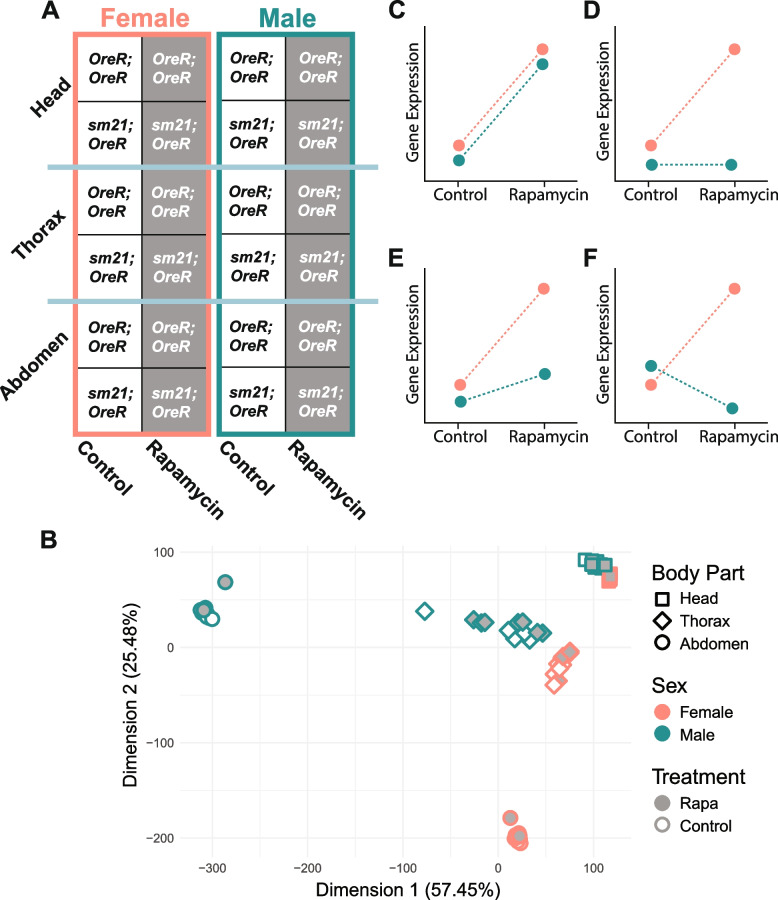
The effect of rapamycin treatment on gene expression. **A** The fully-factorial experimental design used to assess the transcriptomic response to rapamycin in every combination of sex, tissue, and mtDNA. **B** Multidimensional scaling (MDS) analysis of the 71 transcriptomes in the study following variance-stabilizing transformation. These data include all detected transcripts without regard to the statistical significance of differential expression. **C-F** Schematic representations of possible transcriptional responses to rapamycin. **C** An example of a transcript exhibiting no interaction between rapamycin and another factor (here, sex): both males and females respond to rapamycin in a coordinated manner. **D, E, F** Examples of transcripts exhibiting sex-by-treatment interactions. **D** rapamycin affects expression in only one sex, **E** the magnitude of the response to rapamycin differs between sexes, and **F** the direction of the response to rapamycin differs between sexes

To model disrupted mitonuclear communication, we used a *Drosophila* mitochondrial introgression strain, bearing the nuclear genome of *D. melanogaster* line *OregonR* and the ‘foreign’ mitochondrial genome from a closely related *Drosophila* species, *D. simulans*. To model intact mitonuclear communication, we compared this introgression strain to the isogenic *D. melanogaster OregonR* strain bearing its native mtDNA. This experimental system has been described in several earlier studies [38, 40–42]. Hereafter, we denote the *D. melanogaster* line as *OreR*;*OreR* (*OregonR* mtDNA paired with *OregonR* nuclear genome) and the mitochondrial introgression line as *sm21*;*OreR* (*D. simulans* line *sm21* mtDNA paired with *OregonR* nuclear genome). In this study, we subjected *OreR*;*OreR* and *sm21*;*OreR* adult flies, first segregated by sex, to a three-day regimen of either rapamycin or control food (see Methods). Following the treatment period, total RNA was isolated from three body parts (or, tissues for simplicity) – head, thorax, and abdomen – and sequenced in a fully factorial design (Fig. 1A; the read count table is available as Supplementary Table S1). We note that in terms of biomass, heads are primarily composed of neural tissue, thoraces are primarily composed of muscle tissue, and abdomens are a heterogeneous mix of gonad, muscle, fat body, and gut; cuticle tissue is present in all body parts. Initial comparative analysis of the transcriptomes confirmed substantial variability among the sexes and across the three tissues (Fig. 1B), with relatively minor differences between treatments and strains of *Drosophila* (Fig. 1B; see MDS analyses of individual body parts in Fig. S1 for the comparison of transcriptomes between mtDNAs).

Our experimental design enables us to quantify the main first-order effects of rapamycin on gene expression in different clinically relevant contexts – i.e., to identify genes differentially expressed (DE) by rapamycin in different sexes or tissues. We also model the two-way interactions between rapamycin treatment and the other three experimental factors to identify genes with significant interaction (second-order) effects of sex, tissue, and mtDNA on the transcriptomic response to rapamycin. In brief, transcripts that lack significant interaction terms have broadly concordant patterns of expression across treatments (control and rapamycin) at the different levels of another factor (sex, tissue, or mtDNA). For instance, genes exhibiting no interaction between treatment and sex respond to rapamycin similarly in both males and females (Fig. 1C). In contrast, genes with significant interaction terms are affected by rapamycin differently across the levels of another factor, suggesting the influence of the latter on the transcriptomic response to rapamycin. For example, genes exhibiting a significant sex-by-treatment interaction may respond to rapamycin in one sex but not in the other (e.g., Fig. 1D; see Figs. 1E and 1F for visualizations of other possible types of interactions).

### Main effects of rapamycin in female and male tissues

**Fig. 2:**
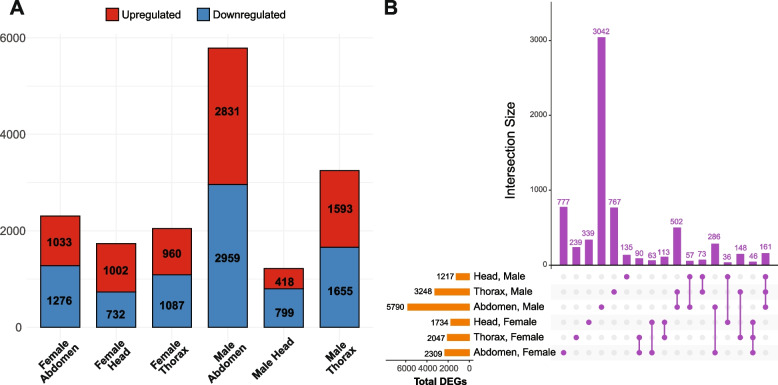
First-order rapamycin effects on gene expression. **A** Counts of genes upregulated and downregulated by rapamycin in each sex-tissue combination. **B** Intersections between genes DE by rapamycin in different sex-tissue combinations show a considerable number of unique DEGs (compare the single dots to the paired dots in the UpSet plot)

To evaluate the first-order ‘main’ effects of rapamycin on gene expression, we partitioned the dataset into six sex-tissue combinations (Fig. 1A). To assess differential expression in each sex-tissue combination, we used the Wald test as implemented in DESeq2 [43] and a design that accounts for the rapamycin treatment while controlling for the differences between mitochondrial genotypes: Expression ~ Treatment + mtDNA. Our analysis revealed a substantial number of transcripts DE due to the rapamycin treatment (at FDR < 0.05) in each sex-tissue combination: 5790 in the male abdomen, 1217 in the male head, 3248 in the male thorax, 2309 in the female abdomen, 1734 in the female head, and 2047 in the female thorax (Supplementary Table S2). The counts of up- and down-regulated genes were comparable in each pairing (Fig. 2A). We found that a number of differentially expressed genes (DEGs) were shared between different tissues within each sex and between same tissues across sexes (Fig. 2B). However, each sex-tissue combination also had a considerable fraction of unique DEGs, highlighting the differential effects of rapamycin between the sexes and tissues studied (Fig. 2B). To assess the functional consequences of rapamycin treatment, gene ontology (GO) enrichment analyses were performed on each set of DEGs. In line with prior research, genes differentially expressed due to rapamycin treatment were enriched for GO categories associated with mTORC1 signaling. These included ribosome biogenesis, rRNA processing, metabolism, cell growth, and development (thorax: Fig. S2; head: Fig. S3; abdomen: Fig. S4; Supplementary Table S3).

### Sex-by-rapamycin effects in each body part

To identify genes with significant sex-by-treatment interaction effects (i.e., genes with a response to rapamycin modulated by sex), we partitioned our data into three body parts (i.e. into three groups represented by rows of Fig. 1A). For the analysis of each group in DESeq2, we used a design formula that includes the effects of sex, rapamycin treatment, mtDNA, and all their interactions: Expression ~ Sex + Treatment + mtDNA + (Sex × Treatment) + (Sex × mtDNA) + (Treatment × mtDNA) + (Sex × Treatment × mtDNA).

**Fig. 3:**
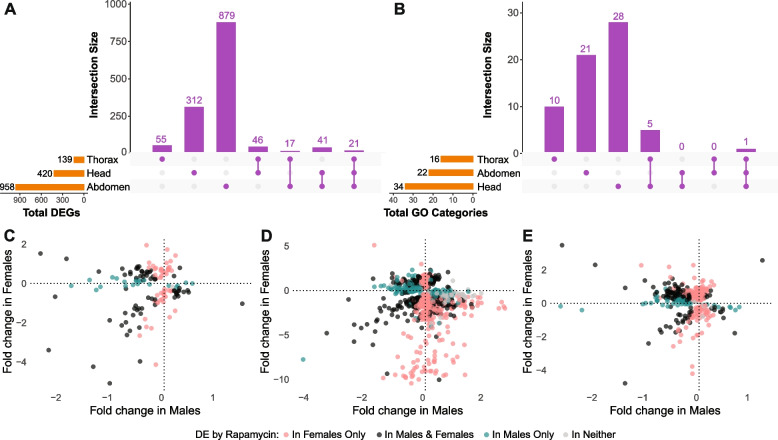
Sex-by-treatment interactions in gene expression in different tissues. **A** The majority of DEGs sensitive to sex-treatment interaction in the abdomen and head, as well as the plurality of DEGs in the thorax are private to the respective tissue. **B** Enriched GO categories exhibit minimal overlap between tissues. **C, D, E** Estimated log_2_ fold change in response to rapamycin (as a first-order effect of a contrast between rapamycin and control conditions, analysis in Fig. 2) in males against the log_2_ fold change estimated in females for every DEG exhibiting significant sex-by-treatment interaction in thorax (**C**), abdomen (**D**), and head (**E**). Note that for the sex-by-treatment DEGs in top-right and bottom-left quadrants of each panel, sex modulates the magnitude of the response to rapamycin. In the top-left corner of each panel, DEGs are upregulated by rapamycin in females and downregulated in males (as conceptualized in Fig. 1F). In the bottom-right corner of each panel, DEGs are upregulated in males but downregulated in females (the inverse of Fig. 1F)

We identified a substantial number of genes with significant sex-by-treatment interaction terms: 958 in abdomen, 420 in head, and 139 in thorax (Fig. 3A; Supplementary Table S4). Of these genes, some were shared across the three tissues (21 shared by all three, 62 between the abdomen and head, 38 between the thorax and abdomen, and 67 between the head and thorax). However, a substantial proportion of DEGs were exclusive to each specific body part: 91.7% of DEGs (879/958) in the abdomen, 39.5% (55/139) in the thorax, and 74.3% (312/420) in the head, indicating tissue-specific modulation of the rapamycin response by sex (Fig. 3A). To gain further insight into the nature of sex-by-treatment interaction in each tissue, we compared the first-order effects of rapamycin in males and females (calculated in Fig. 2) for all genes sensitive to the sex-by-treatment interaction (Supplementary Table S4). We observed all three types of sex-by-treatment interactions represented in Fig. 1 in each of the body parts studied (Figs. 3C, 3D, and 3E). Firstly, we found a considerable number of genes with a significant response to rapamycin in one sex only (pink and teal dots in Fig. 3D corresponding to the example in Fig. 1D). We also found genes for which sex modulated the magnitude of response to rapamycin (those in the top-right and bottom-left quadrants in Figs. 3C, 3D, and 3E, corresponding to Fig. 1E). Finally, we identified many genes with inverted responses to rapamycin in the two sexes (i.e., upregulated in one sex and downregulated in the other; see the top-left and bottom-right quadrants in Figs. 3C, 3D, and 3E) – or, in other words, genes for which sex modulated the actual direction of response to rapamycin, as illustrated in Fig. 1F.

**Fig. 4:**
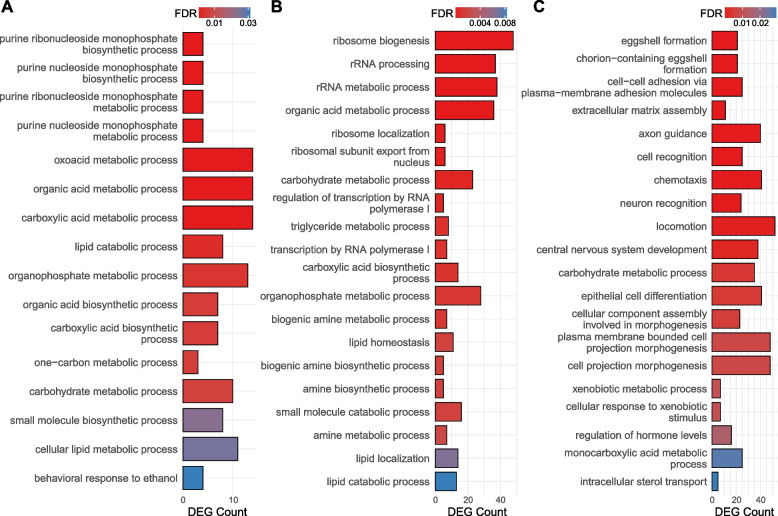
GO enrichment analysis of the sex-by-treatment interaction. Biological processes enriched among the DEGs sensitive to sex-treatment interaction in **A** Thorax, **B** Head, and **C** Abdomen. Top 20 categories by lowest FDR. All results in Supplementary Table S5

To investigate the function of genes differentially expressed by the sex-by-treatment interaction, we conducted gene ontology enrichment analysis, as well as pathway enrichment analysis using the KEGG (Kyoto Encyclopedia of Genes and Genomes) pathway database, and gene set enrichment analysis (GSEA). (The results are available in Supplementary Table S5.) Consistent with many of the DEGs being specific to an individual tissue, we observe minimal overlap among enriched GO categories among different body parts (Fig. 3B). In the thorax – the tissue with the fewest DEGs – we find the vast majority of enriched GO categories to be associated with purine metabolism, purine biosynthesis, and lipid and carbohydrate metabolism (Fig. 4A). DEGs with significant interaction terms in the head are enriched for a greater variety of functional categories, including ribosome biogenesis and localization, rRNA processing, different metabolic processes, cell growth, and transcription (Fig. 4B). Finally, in the abdomen – the tissue with the most genes DE by sex-treatment interaction – we find a large number of processes associated with egg production among enriched functional categories (Fig. 4C). In addition, the abdomen shows enrichment in biological processes related to nervous system development, axon guidance, cell recognition, cell–cell adhesion, extracellular matrix assembly, intracellular sterol transport, some metabolic processes, and the response to xenobiotic stimuli. We note that the abdomen samples include the ovaries or testes plus a heterogeneous mixture of other cell types with significant physiological differences between sexes. This likely accounts for the large and diverse set of DEGs sensitive to the sex-by-treatment interactions. All in all, our analysis revealed that genes differentially expressed by the sex-treatment interaction were functionally distinct across the three tissues. This conclusion is recapitulated (albeit with fewer enriched categories) by the KEGG pathway enrichment analysis (Fig. S5) and GSEA (Fig. S6).

### Tissue-by-rapamycin effects in each sex

To assess the influence of tissue on the transcriptional response to rapamycin, we partitioned our data by sex (corresponding to the columns of Fig. 1A). Then, within each sex, we assayed differential expression using a design accounting for the effects of tissue, rapamycin treatment, and mitonuclear genotype (and their interactions) as follows: Expression ~ Tissue + Treatment + mtDNA + (Tissue × Treatment) + (Tissue × mtDNA) + (Treatment × mtDNA) + (Tissue × Treatment × mtDNA).

**Fig. 5:**
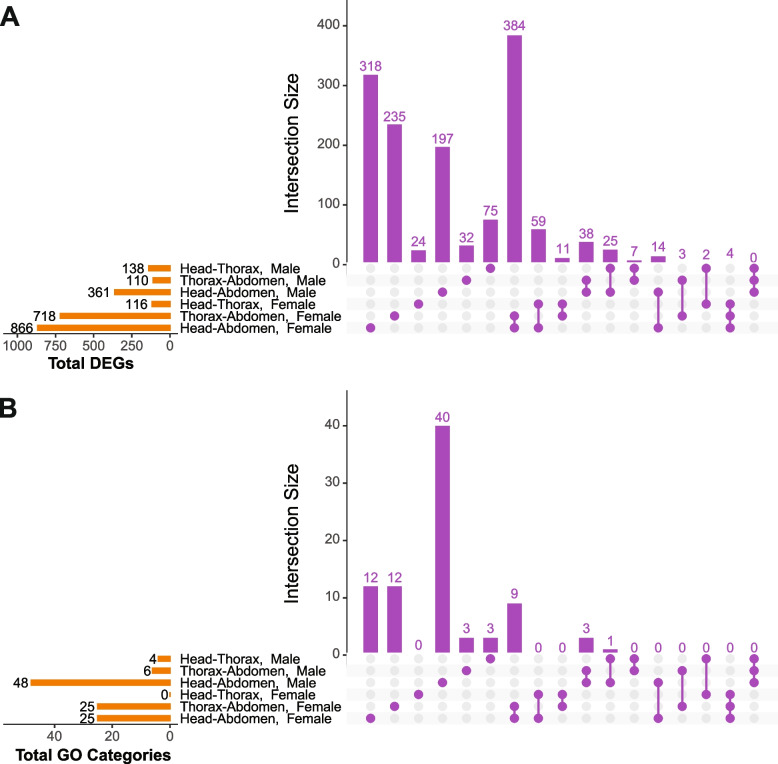
Tissue effects on rapamycin response. **A** Total DEGs (orange bars) sensitive to tissue-treatment interactions for each possible combination of two tissues and their notable intersections (purple bars). **B** Enriched GO categories exhibit minimal overlap between tissue combinations or sexes

Again, we identified a large number of genes with significant tissue-by-treatment interaction terms (Fig. 5A, Supplementary Table S7). Because our experimental design included three different body parts (i.e., tissues), we were able to detect genes differentially expressed by the tissue-by-treatment interaction for each pair of tissues (i.e., head vs. thorax, thorax vs. abdomen, and abdomen vs. head). We find most DEGs sensitive to the tissue-treatment interaction when comparing female abdomen vs. thorax and female abdomen vs. head (866 and 718, respectively), with a substantial overlap in differentially expressed genes. In males, we find considerably fewer genes differentially expressed by the tissue-by-treatment interaction in the abdomen vs. thorax and abdomen vs. head comparisons (110 and 361, respectively). Finally, comparisons between the head and thorax in both males and females showed a moderate number of DEGs with tissue-specific treatment effects (138 for males and 116 for females).

**Fig. 6:**
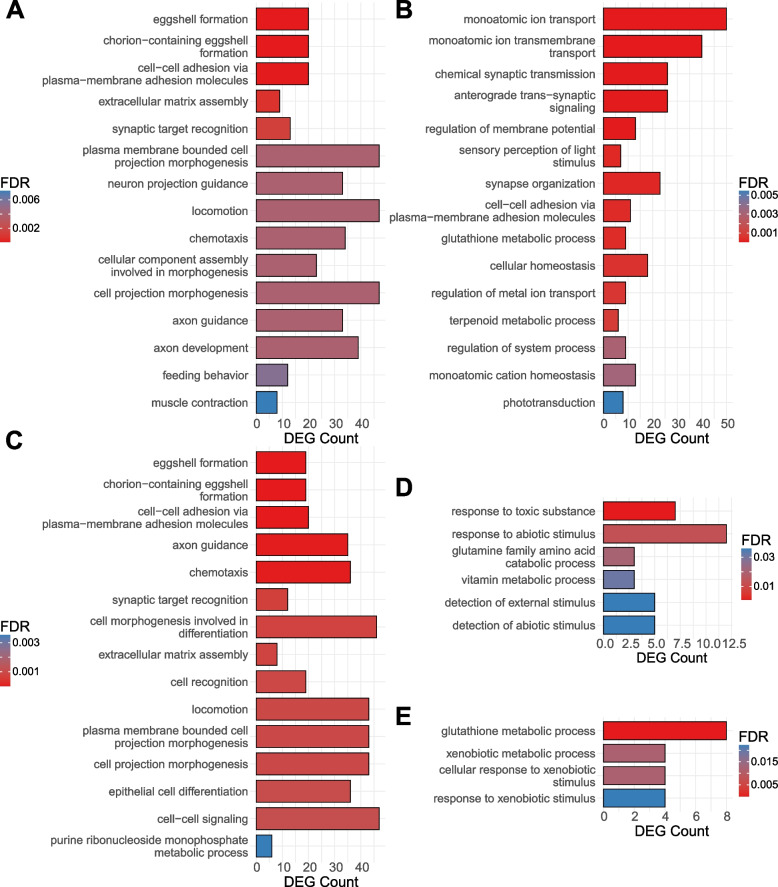
GO enrichment analysis of the tissue-by-treatment interaction. Biological processes enriched among the DEGs sensitive to sex-treatment interaction between **A** Female Abdomen and Head, **B** Male Abdomen and Head, **C** Female Abdomen and Thorax, **D** Male Abdomen and Thorax, and **E** Male Thorax and Head. Top 15 (or fewer) categories by lowest FDR. All results in Supplementary Table S8

To investigate the biological roles of genes sensitive to tissue-by-treatment interactions, we again conducted GO enrichment analyses. We find minimal overlap between GO categories enriched in most tissue combinations except in abdomen-related contrasts (Fig. 5B). Among DEGs sensitive to tissue-by-treatment interactions in female abdomen vs. head and abdomen vs. thorax comparisons, we find enrichment in a number of categories related to eggshell formation, cell–cell adhesion and signaling, extracellular matrix assembly, monophosphate metabolic process, axon guidance, and locomotion (Fig. 6). Interestingly, no GO category enrichment was observed among the genes sensitive to tissue-by-treatment interaction in female head vs. thorax comparisons.

In males, we identified most GO categories enriched among the DEGs sensitive to tissue-by-treatment interaction in the abdomen vs. head contrast and considerably fewer GO categories in the abdomen vs. thorax comparison (Fig. 6). Both comparisons share some categories related to response to abiotic stimulus, though. Moreover, the abdomen vs. head comparison revealed DEGs exhibiting tissue-by-treatment interaction to be enriched for a number of categories associated with ion transport, cell–cell adhesion and signaling, phototransduction, axonogenesis, and synapse organization. Notably, DEGs from both abdomen-related comparisons in males are enriched for different functional categories than those in females. Furthermore, unlike the corresponding female comparison, limited GO category enrichment was also observed among tissue-by-treatment sensitive DEGs in the male thorax vs. head comparison, related to glutathione metabolic process and responses to xenobiotic stimulus.

### mtDNA genotype-by-rapamycin effects in each tissue and sex

**Fig. 7:**
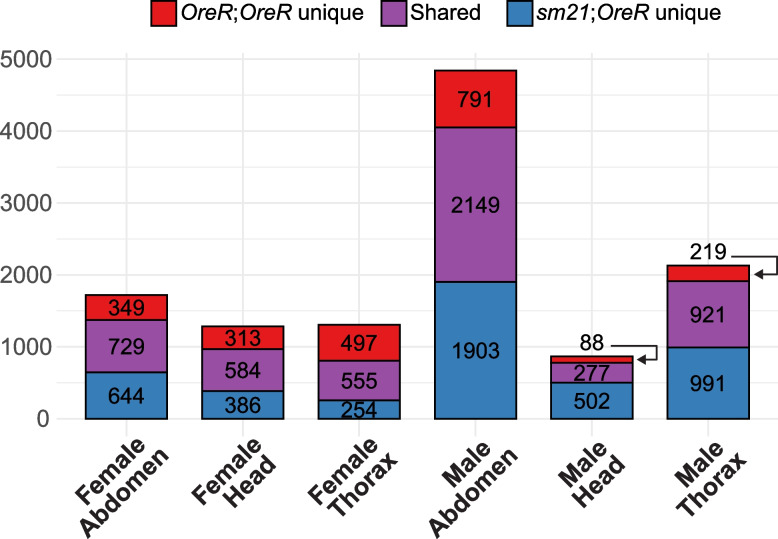
Genes DE by rapamycin in tissues of *OreR*;*OreR* and *sm21*;*OreR* lines. Mitonuclear genotypes share a large fraction of genes DE by rapamycin (purple), yet each has hundreds of private DEGs as well (red and blue)

Next, motivated by the observed differences in the rapamycin transcriptomic response between *OreR*;*OreR* and *sm21*;*OreR* in the earlier study of Santiago et al. [40], we explored the effect of mitochondrial genotype on rapamycin response in our dataset. First, we identified genes with a significant first-order effect of rapamycin on expression in *OreR*;*OreR* and *sm21*;*OreR* flies for each of the six sex-tissue combinations (Supplementary Table S8). (This analysis is similar to that summarized in Fig. 2 above, except rapamycin and control treatments are contrasted within each of the mitonuclear genotypes.) As illustrated in Fig. 7, we find substantial overlap in DEGs sensitive to rapamycin between *OreR*;*OreR* and *sm21*;*OreR* lines for each sex-tissue combination. Despite this overlap, we also detect hundreds of DEGs that are unique for individual mitonuclear genotypes. Overall, though, GO enrichment analysis of DEGs sensitive to rapamycin treatment indicates enrichment for broadly similar functional categories in both *OreR*;*OreR* and *sm21*;*OreR* (females: Fig. S7, males: Fig. S8, results: Supplementary Table S9).

We then looked for DEGs sensitive to the mtDNA-by-treatment interaction within each of the six sex-tissue combinations using a design that includes the effects of treatment, mtDNA, and their interaction: Expression ~ Treatment + mtDNA + (Treatment × mtDNA). We identified a limited number of genes with significant mtDNA-by-treatment interaction terms. We found no DEGs sensitive to mtDNA-treatment interaction in the female abdomen, one gene in the female head, and two in the female thorax. In the male cohort, we detected no DEGs in the thorax, six DEGs exhibiting mtDNA-by-treatment interactions in the male head, and 15 genes in the abdomen (Supplementary Table S10). None of the tissues showed any significant GO category enrichment (with more than two DEGs per category).

Finally, we re-analyzed the transcriptomic response to rapamycin in the recent study of Santiago et al., who subjected the same *OreR*;*OreR* and *sm21*;*OreR* strains as used in the current work to several hours of rapamycin or control food following an overnight starvation period. RNA was isolated and sequenced from male eviscerated abdomen after 0 (i.e., at the start), 1, 2, and 4 h of feeding (rather than 3 days). Santiago et al. explored gene expression in response to rapamycin across the course of the experiment to identify gene clusters with inverted expression patterns between the *OreR*;*OreR* and *sm21*;*OreR* strains, particularly evident after the 4 h rapamycin treatment (refer to Fig. 3B in [40]).

**Fig. 8:**
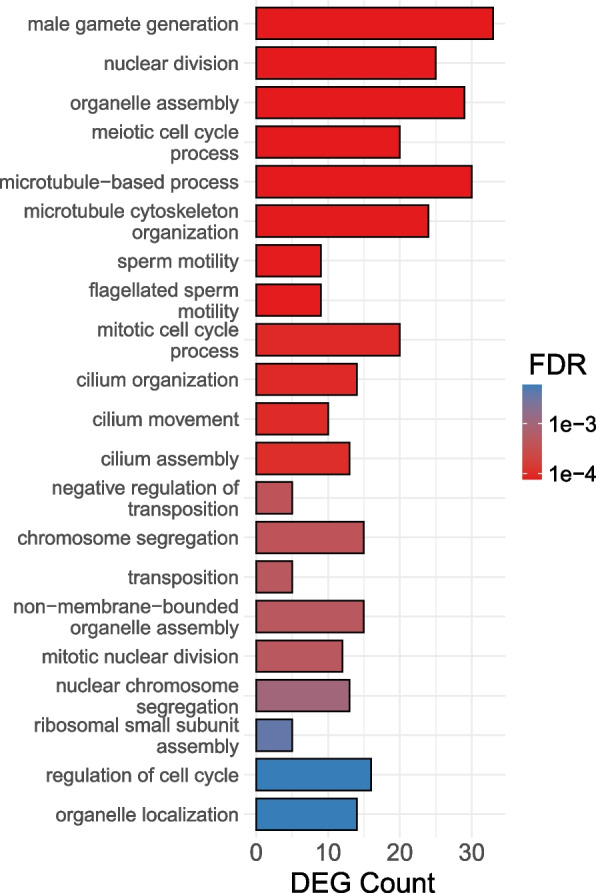
GO enrichment analysis of the mtDNA-by-treatment interactions in Santiago et al. (2021). Reanalysis of the data from Santiago et al. revealed similar functional enrichment among DEGs sensitive to mtDNA-by-treatment interactions and the clusters of genes identified by the authors. All results in Supplementary Table S11

To formally investigate mtDNA-by-treatment interactions in the Santiago et al. study, we partitioned the expression data (available at NCBI SRA, BioProject accession: PRJNA610872) into the three individual time points following refeeding: 1 h, 2 h, and 4 h. At each time point we identified genes with significant mtDNA-by-treatment interaction terms as above. Few DEGs were sensitive to the mtDNA-by-treatment interaction at 1 and 2 h of refeeding (5 and 7 genes respectively). However, we detected a considerable number of DEGs exhibiting mtDNA-by-treatment interactions at 4 h (*n* = 251, Supplementary Table S11), consistent with the inversion in temporal expression patterns observed by Santiago et al. at the same time point. Functional enrichment analysis of interaction-sensitive DEGs revealed GO categories associated with meiotic cell cycle processes and sperm generation and motility (possibly indicating the presence of some gonad tissue in the eviscerated abdomen), as well as nuclear division, and organelle assembly (Fig. 8; Supplementary Table S11).

## Discussion

In this study, we have explored how sex, tissue type, and mitonuclear genotype influence the transcriptional response to rapamycin. To do so, we assayed gene expression following rapamycin treatment in three distinct tissues of fruit flies bearing either their native or foreign mtDNA. Utilizing a fully-factorial experimental design, we analyzed both the main effects of rapamycin and its interactions with other experimental factors. We have focused our analysis on genes with significant sex-by-treatment, tissue-by-treatment, and mtDNA-by-treatment interaction terms because they respond differentially to rapamycin across sexes, tissues, and mitochondrial backgrounds. For example, genes exhibiting tissue-by-treatment interactions may be downregulated by rapamycin in one tissue but upregulated in another (refer to Fig. 1 for visual representations of possible treatment interactions). These genes are of particular interest to us because the variance in rapamycin response may lead to unforeseen side-effects that could compromise the efficacy of mTOR inhibition in the clinic. Our findings reveal treatment interactions to be fairly common (especially the influence of sex and tissue on the response to rapamycin) and affecting a variety of critical biological functions. These findings can guide further studies seeking to refine the efficacy and reduce the detrimental side effects of mTOR inhibitors like rapamycin.

### Sex-specific effects

We found that sex exerted the most pronounced influence on the transcriptional impact of rapamycin, clearly indicating that the outcomes of mTOR-inhibiting therapies may strongly depend on the patient’s sex. The observation that sex has a significant effect on the response to rapamycin aligns well with previous research, which demonstrated sexually dimorphic effects of rapamycin on male and female longevity in *D. melanogaster*, *C. remanei* nematodes, and mice [17, 30–32, 44]. Our investigation also revealed significant differences in the nature of the sex-by-treatment interaction among the three tissues we studied, both in the numbers and identities of differentially expressed genes, as well as the types of biological processes overrepresented among those DEGs.

We detected the fewest differentially expressed genes sensitive to sex-by-treatment interactions in the thorax. This analysis seeks to detect sexually dimorphic effects of rapamycin on gene expression. The thorax is predominantly composed of muscles, powering flight, movement, and courtship songs – a male-specific behavior used to attract mates in which a wing is vibrated to produce a series of pulses and tones [45]. Within the thorax, we observed sex to modulate mostly the metabolic response to rapamycin with a particularly strong influence on rapamycin’s effect on purine metabolism. The latter observation is consistent with previous studies, which have demonstrated that mTORC1 promotes purine biosynthesis [46], while purine availability, in turn, regulates mTORC1 activity [47]. It is notable that purine metabolism is affected in the muscle specifically, where the purine nucleotide cycle serves as a crucial metabolic pathway for replenishing ATP reserves from purine adenosine monophosphate (AMP) following physical activity or periods of starvation. Our finding that purine metabolism in the muscle appears to exhibit sex-by-treatment interaction points to differing, sex-dependent effects of rapamycin on this key cycle of ATP reservoir recovery in muscles. We also note that, while these sex-by-rapamycin effects on purine metabolism genes make sense, only one of the four primary flight muscles is larger in males than in females [39], which may explain why this tissue type had the fewest sex-specific DEGs showing rapamycin interaction effects.

We observed marked differences in the influence of sex on the rapamycin response in the head and in the thorax, consistent with the distinct roles of mTOR signaling in brain and nerve tissue compared to muscle. mTOR has been implicated in a number of processes critical for proper brain function, including regulating neural development, circuit formation, and autophagy (which is thought to protect the brain from neurodegenerative disorders such Parkinson’s and Alzheimer’s diseases [29, 48]). In particular, mTOR signaling is involved in promoting activity-dependent protein translation near synapses, enabling modifications in neural circuits through localized changes in synaptic structure and function [29, 49, 50]. Intriguingly, we found DEGs sensitive to sex-by-treatment interaction in the head to be enriched for ribosome biogenesis, rRNA processing, and ribosome localization – processes critical for localized protein synthesis – suggesting that the realized effects of rapamycin on synapse formation and maintenance may vary between sexes.

Finally, we identified the most differentially expressed genes sensitive to sex-by-treatment interactions in the abdomen (958 out of a total of 20,453 transcripts with recorded expression). The abdomen is, notably, a more heterogeneous region of the fly body compared to the head or thorax. It contains several different tissues, including muscle, heart, the digestive system, the fat body, and the reproductive organs. This heterogeneity likely contributes to the greater prevalence of rapamycin effects and the diversity of sex-by-treatment interactions observed. Most obviously, the abdomen houses the reproductive system, where mTOR signaling is expected to perform a variety of different functions in males and females [51]. For example, mTOR signaling is required in female ovaries to promote egg chamber development in flies [52, 53]. Correspondingly, rapamycin has been shown to interfere with egg production [54], which can explain the enrichment of egg development-related processes we found among genes DE by rapamycin in the female abdomen (Fig. S4A) and genes DE by sex-by-treatment interaction in the abdomen overall (Fig. 4).

An intriguing recent study in *D. melanogaster* demonstrated that rapamycin attenuated intestinal aging in female flies by upregulating autophagy in enterocytes but had no effect in males [32]. Instead, male enterocytes appear to have intrinsically higher levels of autophagy that were not further increased by rapamycin. The authors measured autophagy by quantifying the presence of the lipidated form of the Atg8a protein at the age of 10 days, showing that rapamycin increased Atg8a levels in females but not males. In contrast, we did not detect Atg8a, or any of the Atg genes, among those exhibiting sex-by-treatment interactions in the abdomen. We note, though, that flies in our study were treated with rapamycin for a shorter period than in [32] – 3 days vs. 10 days – potentially accounting for the difference in the results.

### Tissue specific effects

Our analysis revealed that body part is also a significant modifier of the transcriptional impact of rapamycin, suggesting that therapeutic interventions targeting mTOR signaling may have significantly different effects in individual tissues, which must be accounted for in the clinic. The nature of tissue-by-treatment interactions we discovered was contingent on the tissues being compared. We found the highest number of differentially expressed genes sensitive to tissue-by-treatment interactions when comparing the abdomen to either head or thorax in females. (Meanwhile, the head-thorax comparison in females yielded considerably fewer DEGs, with no significant functional enrichment). Some of the enriched categories aligned well with our understanding of the role of mTOR signaling in the abdomen versus the other two tissues. In both abdomen-thorax and abdomen-head comparisons in females, DEGs sensitive to the tissue-by-treatment interaction were enriched for processes related to egg production, consistent with decreased fecundity previously observed in female *D. melanogaster* exposed to rapamycin [55]. Additionally, a considerable number of genes exhibiting tissue-by-treatment interactions in both abdomen-head and abdomen-thorax comparisons were involved in cell–cell adhesion. Notably, we also observed an enrichment of adhesion-related genes among DEGs sensitive to the sex-by-treatment interaction in the abdomen. Cell adhesion has been previously shown to be regulated by mTOR activity [56, 57], but the mechanism of this regulation remains incompletely understood.

The prevalence and type of tissue-by-treatment interactions observed in parallel comparisons among males were different from those among females. Overall, we detected fewer DEGs sensitive to the tissue-by-treatment interactions in males than in females, suggesting that, at least in our system, male tissues are less likely to respond differentially to rapamycin treatment than female tissues. We also found tissue-by-treatment interactions to be functionally distinct in male and female tissues, with no overlap between GO categories enriched in tissue-by-treatment DEGs in male and female contrasts (Fig. 5B). Whereas DEGs displaying tissue-by-treatment interactions in the abdomen-head comparison among females are heavily skewed towards egg production, in males we find enrichment for functions closely aligned with the activity of the nervous system, such as trans-synaptic signaling, axonogenesis, axon guidance (also seen in females), and phototransduction. Indeed, a considerable number of tissue-by-treatment DEGs (*n* = 65) in the male abdomen-head contrast appear to be involved in ion transport. Brains, notably, utilize a significant amount of the body's metabolic energy [58, 59] with most of the energy produced in the nervous system used in trans-synaptic signaling [59] to restore ionic concentration gradients at the synapse following depolarization [59, 60]. It makes sense that mTOR inhibition in the head would preferentially affect some of the most energy-consuming functions, pointing to potential side effects for cognition and neuronal function in males on rapamycin treatment.

It is also noteworthy that DEGs exhibiting tissue-by-treatment interactions in both the male abdomen-thorax and male head-thorax contrasts are primarily involved in either the detection and response to abiotic and toxic stimuli or glutathione metabolism (and that of its precursor, glutamine). Intriguingly, glutathione is an effective antioxidant [61] crucial for cells’ ability to cope with oxidative stress. These results suggest that rapamycin may differentially affect the ability of the thorax to manage oxidative stress compared to other fly tissues. Notably, the ability to respond to oxidative stress is potentially most crucial in the thorax, as muscle activity is known to elevate the production of reactive oxygen species [62]. Furthermore, considering that we did not observe the same enrichment pattern in females, it appears that the muscle’s ability (i.e., that of the thorax) to respond to oxidative stress under the influence of rapamycin may also differ between the sexes.

### Mitochondrial genotype effects

We found little signal of mtDNA-by-treatment interaction in any of the tissue-by-sex combinations we analyzed. mTOR is known to play a crucial role in proper mitochondrial function, coordinating mitochondrial respiration, biogenesis (e.g., by stimulating the synthesis of mitochondria-related proteins), and apoptosis [35, 37, 63, 64]. Modulation of mTOR signaling has also shown promise in mitochondrial disfunction. For instance, mTOR inhibition has been found to rescue phenotypes associated with mitochondrial disfunction in yeast [65]. Additionally, rapamycin treatment has been shown to extend survival and alleviate neurological symptoms in a mouse model of Leigh syndrome [66], a childhood mitochondrial disease, and to inhibit progression and improve the condition of mice with mitochondrial myopathy, a common manifestation of adult-onset mitochondrial disease in the muscle [67].

In an earlier study, Santiago et al. hypothesized that mitonuclear communication could, in turn, modulate mTOR signaling pathways by relaying mitochondrial status through retrograde mitochondrial signaling [40]. Indeed, Santiago et al. found clusters of genes where the transcriptional response to rapamycin over several hours of treatment was inverted between the same two *OreR*;*OreR* and *sm21*;*OreR* lines we have used in our study. These finding are consistent with the influence of mtDNA-by-treatment interactions on the response to rapamycin, even though the study was conducted to compare the main effects of rapamycin between mitotypes rather than detect such interactions. We re-analyzed the data from Santiago et al. to statistically test for mtDNA-by-treatment interactions and found several hundred DEGs with significant mtDNA-by-treatment interaction terms (note that Santiago et al. used edgeR [68] to identify DEGs, while we have used DESeq2 [43] in our analyses). Furthermore, we found similar functional enrichment among these DEGs and the clusters of genes with mitonuclear genotype-dependent responses to rapamycin identified by Santiago et al.

Importantly, the design of experiment conducted by Santiago et al. differed significantly from our current study, potentially accounting for the lack of mtDNA-by-treatment interactions in our data. In the earlier study, starved flies were subjected to refeeding with rapamycin for 4 h, whereas in our study, healthy, well-fed flies were given rapamycin-containing food for a considerably longer period of 3 days. It is, thus, possible that the importance of mtDNA-by-treatment interactions changes with the length of the rapamycin treatment and the prior nutritional state of the flies, necessitating a more detailed examination of the temporal transcriptional response to rapamycin in different mitochondrial backgrounds.

## Conclusions

In summary, our study sheds light on the prevalence and complexity of interactions shaping the transcriptional response to rapamycin in fruit flies. Our findings emphasize the significance of sex-specific and tissue-specific rapamycin effects, while also suggesting a potential role of mitonuclear communication that merits further analysis. Above, we have highlighted some of the intriguing sex-by-treatment and tissue-by-treatment interactions of rapamycin. We conclude that understanding the landscape of interactions influencing the response to mTOR inhibition is essential for optimizing therapeutic outcomes of personalized mTOR-targeting therapies and mitigating potential adverse effects.

## Methods

### Fly stocks

To examine the transcriptomic response to rapamycin treatment, we used two lines of *Drosophila* previously described in [38, 40, 41]. * D. melanogaster* line *Oregon R* (*OreR*;*OreR*) was used to model intact mitonuclear communication. The introgression line (*sm21*;*OreR*) representing disrupted mitonuclear communication bears mismatched mitochondrial (mtDNA from a related species *D. simulans* line *sm21*) and nuclear (isogenic *OreR*) genomes. The introgression line was generated (as described in detail in [41]) using balancer chromosome replacement crosses to place *OregonR* chromosomes onto *sm21* cytoplasmic backgrounds. To produce the *sm21*; *OreR* line, female cytoplasm was derived from *D. simulans,* and the nuclear *OreR* chromosomes were introduced by male parents followed by repeatedly backcrossing the *sm21*;*OreR* introgression line to control *OreR*;*OreR* males to produce and maintain isogenic nuclear genomes. All stocks and experiments were maintained under standard conditions (25˚C, 12 h light–dark cycle) on standard laboratory diet (a medium containing 5.2% cornmeal, 2% yeast, 11% sugar and 0.9% agar).

### Rapamycin treatment experiment

Five-day-old, age matched mated flies were separated by sex into cohorts of 30 and transferred to agar vials containing either the standard lab food with 200 μM rapamycin dissolved in ethanol (“Treatment”) or the standard lab food with ethanol added without rapamycin (“Control”). After 3 days, all flies were flash frozen and cryogenically dissected on ice-cold dissection blocks into three body parts: head, abdomen, and thorax. The fly tissues were then placed in chilled TRIzol and homogenized at 30 Hz for 4 min in a TissueLyser (Qiagen). Total RNA was extracted from cell lysates using RNeasy columns (Qiagen). Concentration and contamination were assessed by nanodrop (Thermo Fisher Scientific) analysis with additional quality control by BGI. RNA sequencing was performed by BGI to produce 50 base pair single end reads. BGI pre-processed the raw reads to remove adaptor sequences, contamination and low-quality reads.

### RNA-seq data preprocessing

RNA-seq read quality was assessed using fastqc v0.11.5 [69] and summarized with MultiQC v 1.0 [70]. We used STAR v2.7.10b [71] to align reads in the 2-pass mode. In the first pass,

*OreR*;*OreR* reads were aligned to the Drosophila melanogaster reference genome (version BDGP6.32) obtained from Ensembl, release 109 [72]. *sm21*;*OreR* reads were aligned to the same BDGP6.32 reference genome, with the *sm21 D. simulans* mitochondrion genome sequence [73], GenBank accession number KC244283.1, in place of the *D. melanogaster* mitochondrion genome sequence. In the second pass, reads were mapped again to the respective reference genomes using splice junctions obtained from the first pass. Annotation file for *D. melanogaster OreR*;*OreR* analysis (Drosophila_melanogaster.BDGP6.32.109.gtf) was obtained from Ensembl, release 109. The same annotation was modified for *sm21*;*OreR* analysis by changing the start and stop positions of each gene in the *D. melanogaster* annotation to those in the *D. simulans* reference, KC244283.1. BAM files (sorted by coordinate) generated by STAR were indexed using Samtools v1.16.1 [74] and reads mapping to specific genome features (genes) were counted using featureCounts [75] from the Subread package v2.0.3. The read count data table generated by featureCounts was used for all downstream analyses.

### RNA-seq Data Analysis

Read counts were imported into R statistical software v4.3.0 [76] for further analysis. One of the libraries for female *OreR*;*OreR* heads under control conditions (coincidentally sequenced separately from the other 71 due to issues with the RNA prep quality) was identified as an outlier based on MDS analysis and removed from the study. Dispersion estimation, normalization, and statistical testing for differential expression were performed within DESeq2 package v 1.42.0 [43] using the Wald test under default parameters (lfcThreshold = 0, alpha = 0.05). Independent hypothesis weighting (IHW) was used for *p* values adjustment for multiple hypothesis testing [77]. Significantly differentially expressed genes (DEGs) were identified based on adjusted *p* values (FDR < 0.05) using the Benjamini–Hochberg procedure. Gene ontology (GO) enrichment analysis (focusing on the “biological process” category), KEGG pathway enrichment analysis, and gene set enrichment (GSEA) analysis (again, using GO annotation of biological processes) were performed with R package clusterProfiler [78]. Enriched categories were identified based on adjusted *p* values (FDR < 0.05) using the Benjamini–Hochberg procedure. To calculate enrichment, all genes that were detected in a particular contrast (with at least a single detected transcript among all libraries) were used as background genes. Redundancy of enriched GO terms was reduced with the *simplify* method of the clusterProfiler package under default parameters. GO annotations were obtained from the org.Dm.eg.db annotation package v3.17.0 [79].

### Supplementary Information

Supplementary Material 1: Table S1. Count table. Read count data before normalization.

Supplementary Material 2: Table S2. Rapamycin main effect differential expression analysis. Results of the DESeq2 analysis of contrasts between control and rapamycin treatments for each sex-tissue combination.

Supplementary Material 3: Table S3. GO enrichment analysis of genes differentially expressed by rapamycin as a main effect. Results of the clusterProfiler analyses (enrichGO followed by simplify functions) for each sex-tissue combination.

Supplementary Material 4: Table S4. Sex-by-Treatment effect differential expression analysis. Results of the DESeq2 analysis of the sex-by-treatment interaction for each tissue. Also includes rapamycin main effect log_2_ fold changes from Table S2.

Supplementary Material 5: Table S5. Enrichment analysis of genes with significant Sex-by-Treatment interaction terms. Results of the clusterProfiler analyses (enrichGO followed by simplify function for GO enrichment, enrichKEGG for KEGG enrichment, GSEA function for GSEA) for each tissue.

Supplementary Material 6: Table S6. Tissue-by-Treatment effect differential expression analysis. Results of the DESeq2 analysis of the 3 tissue-by-treatment interactions (head vs. thorax, thorax vs. abdomen, and abdomen vs. head) for each sex.

Supplementary Material 7: Table S7. GO enrichment analysis of genes with significant Tissue-by-Treatment interaction terms. Results of the clusterProfiler analyses (enrichGO followed by simplify functions) for the 3 tissue-by-treatment interactions (head vs. thorax, thorax vs. abdomen, and abdomen vs. head) for each sex.

Supplementary Material 8: Table S8. Rapamycin main effect differential expression analysis separated by mitonuclear genotype. Results of the DESeq2 analysis of contrasts between control and rapamycin treatments for each sex-tissue-mitonuclear genotype combination.

Supplementary Material 9: Table S9. GO enrichment analysis of genes differentially expressed by rapamycin as a main effect separated by mitonuclear genotype. Results of the clusterProfiler analyses (enrichGO followed by simplify functions) for each sex-tissue-mitonuclear genotype combination.

Supplementary Material 10: Table S10. mtDNA-by-Treatment effect differential expression analysis. Results of the DESeq2 analysis of the mtDNA-by-treatment interaction for each sex-tissue combination.

Supplementary Material 11: Table S11. mtDNA-by-Treatment effect differential expression analysis of the Santiago et al. 2021 dataset. Results of the DESeq2 analysis and the clusterProfiler enrichment analysis of the mtDNA-by-treatment interaction after 4 hours of treatment.

Supplementary Material 12: Figure S1. Multidimensional scaling (MDS) analysis of the 71 transcriptomes following variance stabilizing transformation shows little separation by mtDNA. (A) Thorax, (B) Head, (C) Abdomen.

Supplementary Material 13: Figure S2. GO enrichment analysis of the rapamycin main effects in the Thorax. Biological processes enriched among DEGs upregulated (red) and downregulated (blue) by rapamycin in (A) Females, (B) Males (note no enriched categories among upregulated DEGs). Top 10 upregulated and downregulated categories with the lowest FDR. All results in Supplementary Table S3.

Supplementary Material 14: Figure S3. GO enrichment analysis of the rapamycin main effects in the Head. Biological processes enriched among DEGs upregulated (red) and downregulated (blue) by rapamycin in (A) Females, (B) Males. Top 10 upregulated and downregulated categories with the lowest FDR. All results in Supplementary Table S3.

Supplementary Material 15: Figure S4. GO enrichment analysis of the rapamycin main effects in the Abdomen. Biological processes enriched among DEGs upregulated (red) and downregulated (blue) by rapamycin in (A) Females, (B) Males. Top 10 upregulated and downregulated categories with the lowest FDR. All results in Supplementary Table S3.

Supplementary Material 16: Figure S5. KEGG enrichment analysis of the sex-by-treatment interaction. KEGG pathways enriched among the DEGs sensitive to sex-by-treatment interaction in (A) Thorax, (B) Head, and (C Abdomen. All results in Supplementary Table S5.

Supplementary Material 17: Figure S6. Gene set enrichment analysis of the sex-by-treatment interaction. GSEA of GO biological processes among the DEGs sensitive to sex-treatment interaction in (A) Thorax, (B) Head, and (C) Abdomen. All results in Supplementary Table S5.

Supplementary Material 18: Figure S7. GO enrichment analysis of the rapamycin main effects in female *OreR*;*OreR* and *sm21*;*OreR* flies. Biological processes enriched among DEGs upregulated (red) and downregulated (blue) by rapamycin in the head (A: *OreR*;*OreR*; D: *sm21*;*OreR*), thorax (B: *OreR*;*OreR*; E:*sm21*;*OreR*), and abdomen (C: *OreR*;*OreR*; F: *sm21*;*OreR*). Top 10 upregulated and downregulated categories with the lowest FDR. All results in Supplementary Table S9.

Supplementary Material 19: Figure S8. GO enrichment analysis of the rapamycin main effects in male *OreR*;*OreR* and *sm21*;*OreR* flies. Biological processes enriched among DEGs upregulated (red) and downregulated (blue) by rapamycin in the head (A: *OreR*;*OreR*; D: *sm21*;*OreR*), thorax (B: *OreR*;*OreR*; E:*sm21*;*OreR*), and abdomen (C: *OreR*;*OreR*; F: *sm21*;*OreR*). Top 10 upregulated and downregulated categories with the lowest FDR. All results in Supplementary Table S9.

## Data Availability

The raw RNA-seq reads generated in this study have been submitted to the NCBI Sequence Read Archive (SRA) (https://www.ncbi.nlm.nih.gov/sra) under BioProject accession: PRJNA1107595. *D. melanogaster* reference genome is freely available through the Ensembl genome browser (release 109) at https://feb2023.archive.ensembl.org/Drosophila_melanogaster/Info/Index. R scripts used in the analyses are available at https://github.com/yraynes/Fly-Rapa-Interactions.
