# Supplementary figures and images for: Sex, tissue, and mitochondrial interactions modify the transcriptional response to rapamycin in Drosophila
Source: BMC Genomics. 2024 Aug 7;25:766. doi: 10.1186/s12864-024-10647-x (PMC11304892; doi:10.1186/s12864-024-10647-x)

**A**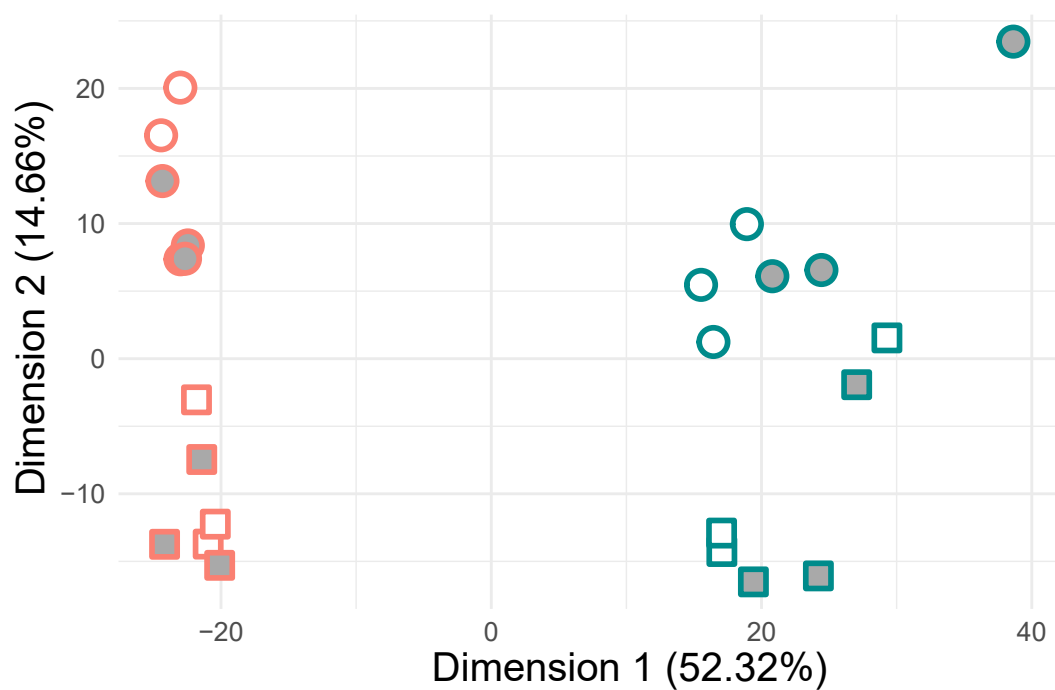**B**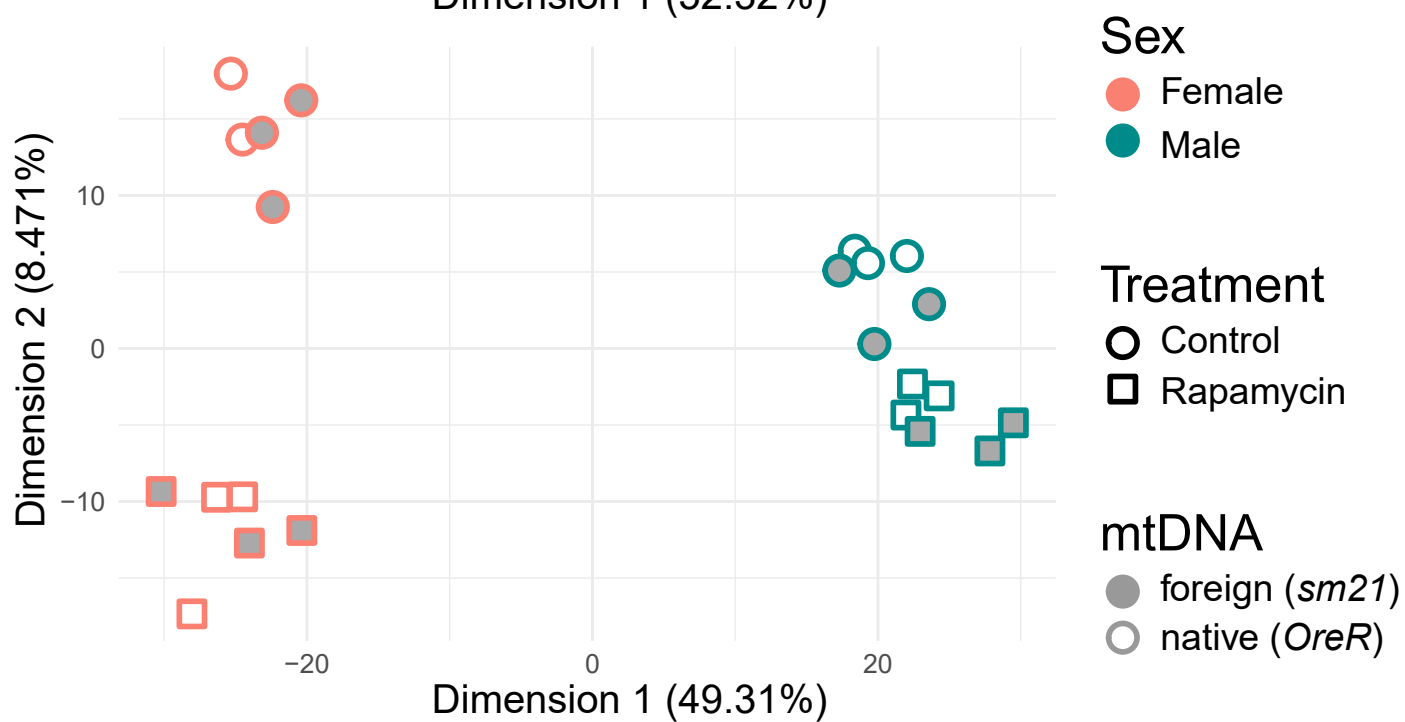**C**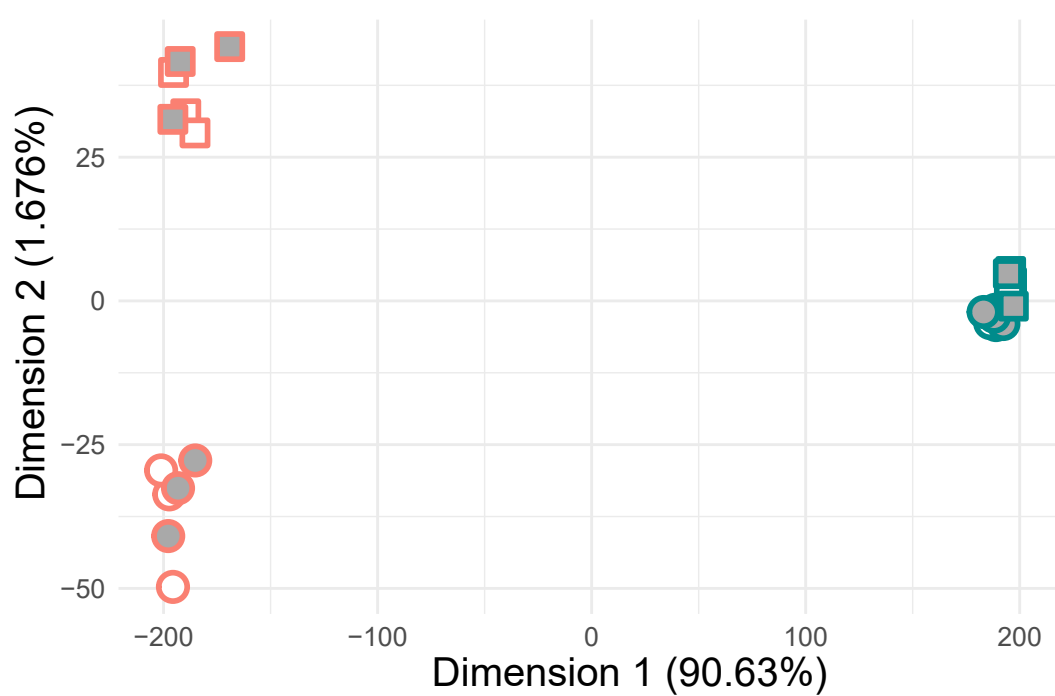

Supplement: Supplementary file 12 — Supplementary Material 12: Figure S1. Multidimensional scaling (MDS) analysis of the 71 transcriptomes following variance stabilizing transformation shows little separation by mtDNA. (A) Thorax, (B) Head, (C) Abdomen. [file 12864_2024_10647_MOESM12_ESM.pdf]

**A**

Ontology

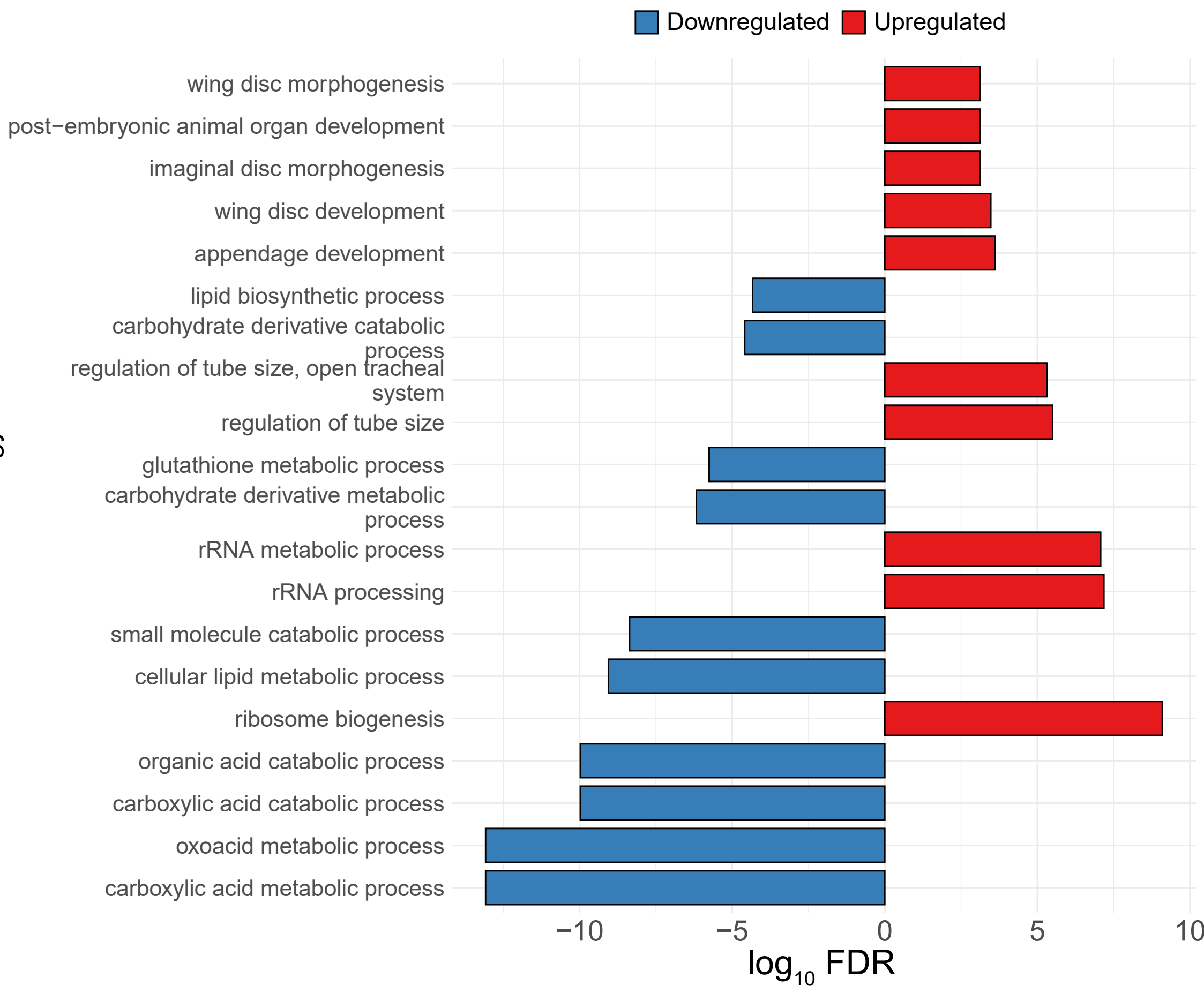**B**

Ontology

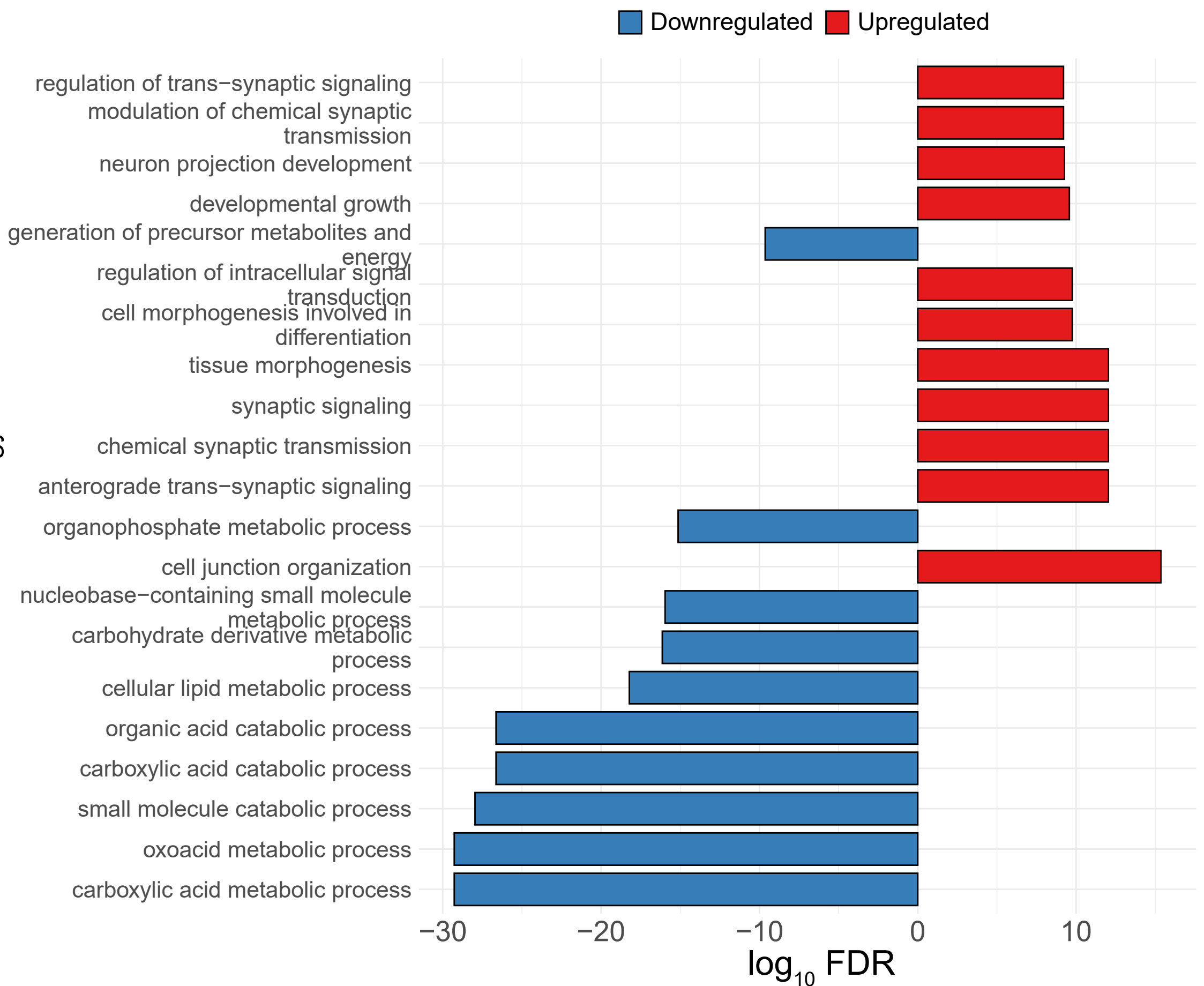

Supplement: Supplementary file 13 — Supplementary Material 13: Figure S2. GO enrichment analysis of the rapamycin main effects in the Thorax. Biological processes enriched among DEGs upregulated (red) and downregulated (blue) by rapamycin in (A) Females, (B) Males (note no enriched categories among upregulated DEGs). Top 10 upregulated and downregulated categories with the lowest FDR. All results in Supplementary Table S3. [file 12864_2024_10647_MOESM13_ESM.pdf]

**A**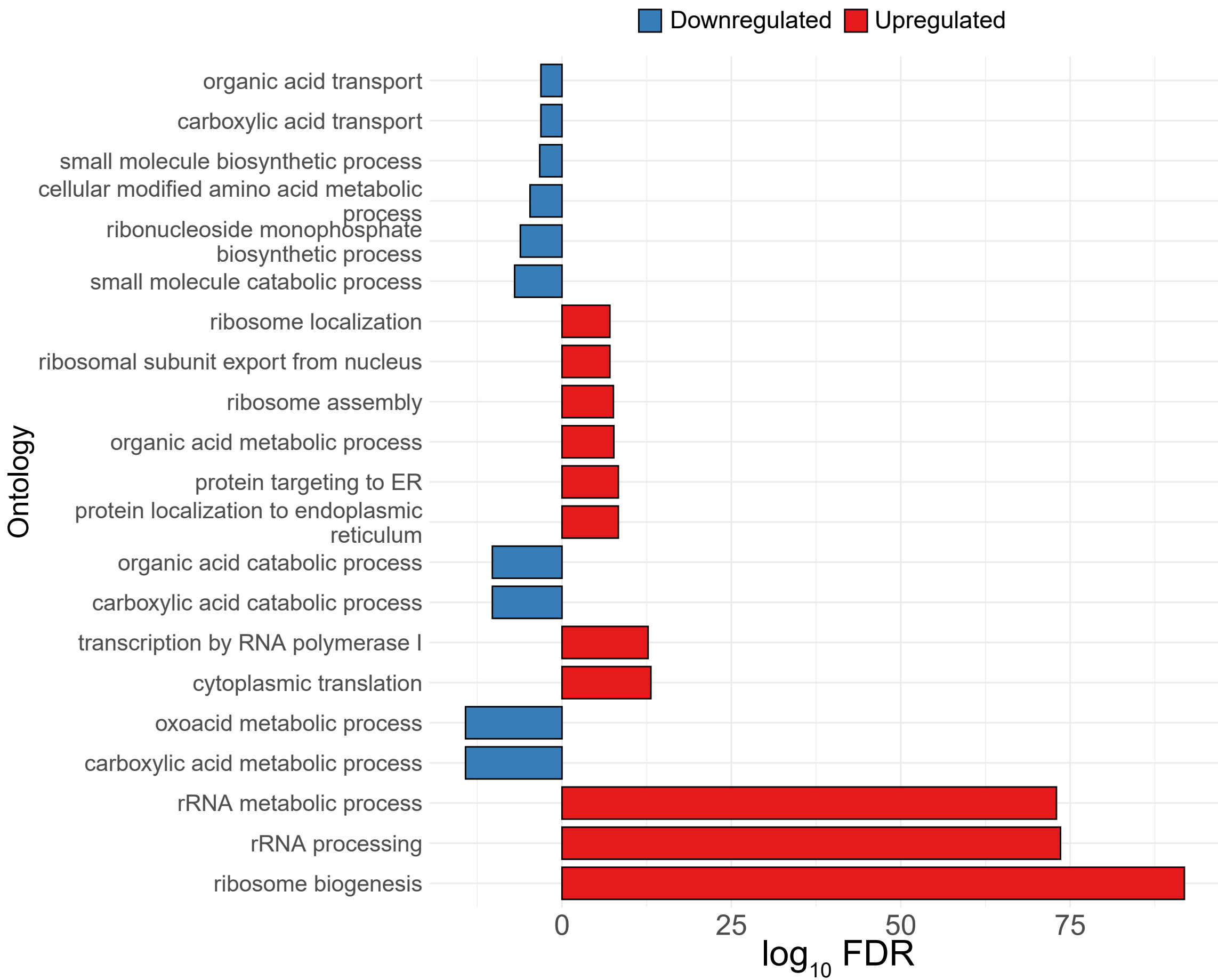**B**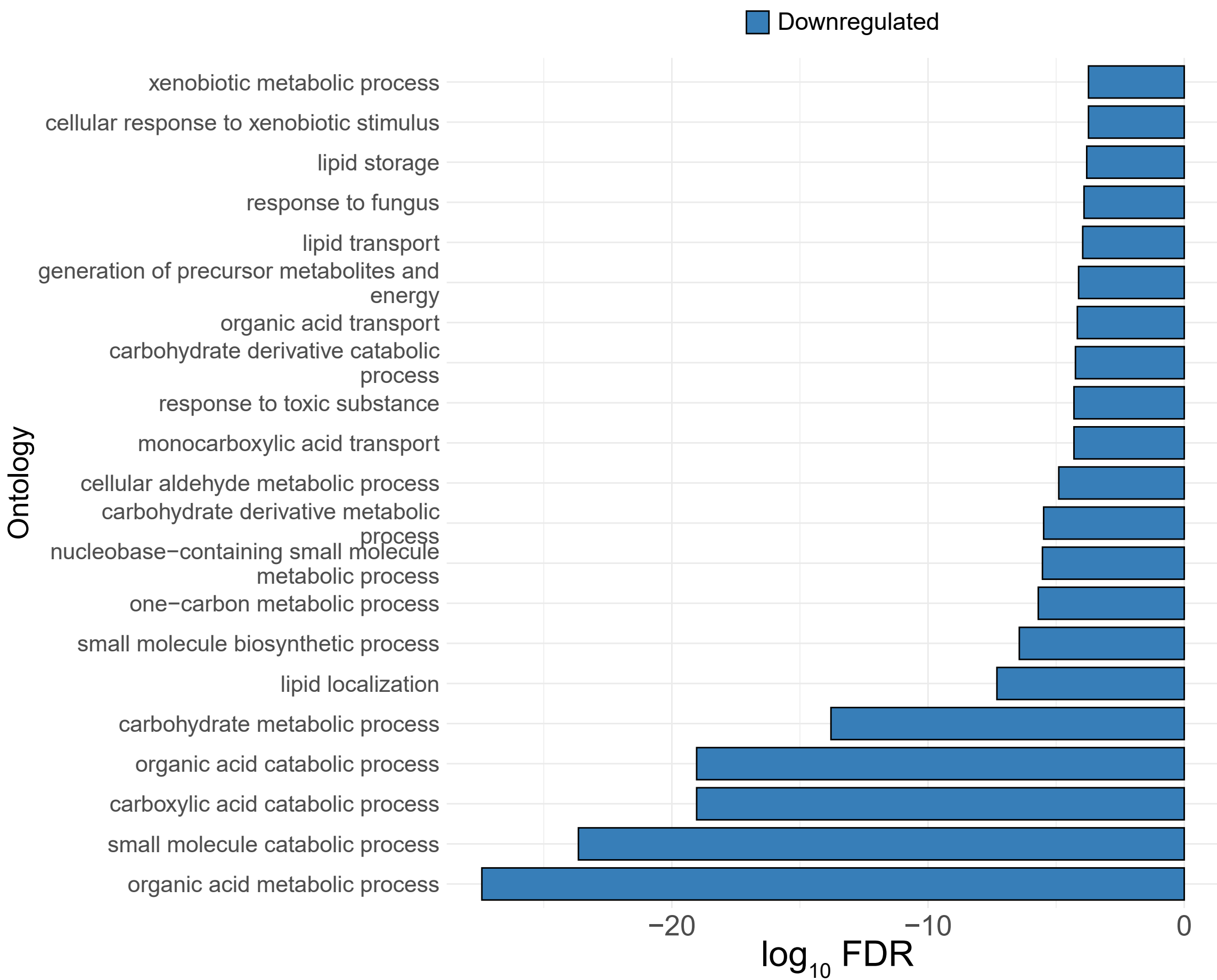

Supplement: Supplementary file 14 — Supplementary Material 14: Figure S3. GO enrichment analysis of the rapamycin main effects in the Head. Biological processes enriched among DEGs upregulated (red) and downregulated (blue) by rapamycin in (A) Females, (B) Males. Top 10 upregulated and downregulated categories with the lowest FDR. All results in Supplementary Table S3. [file 12864_2024_10647_MOESM14_ESM.pdf]

**A**

Ontology

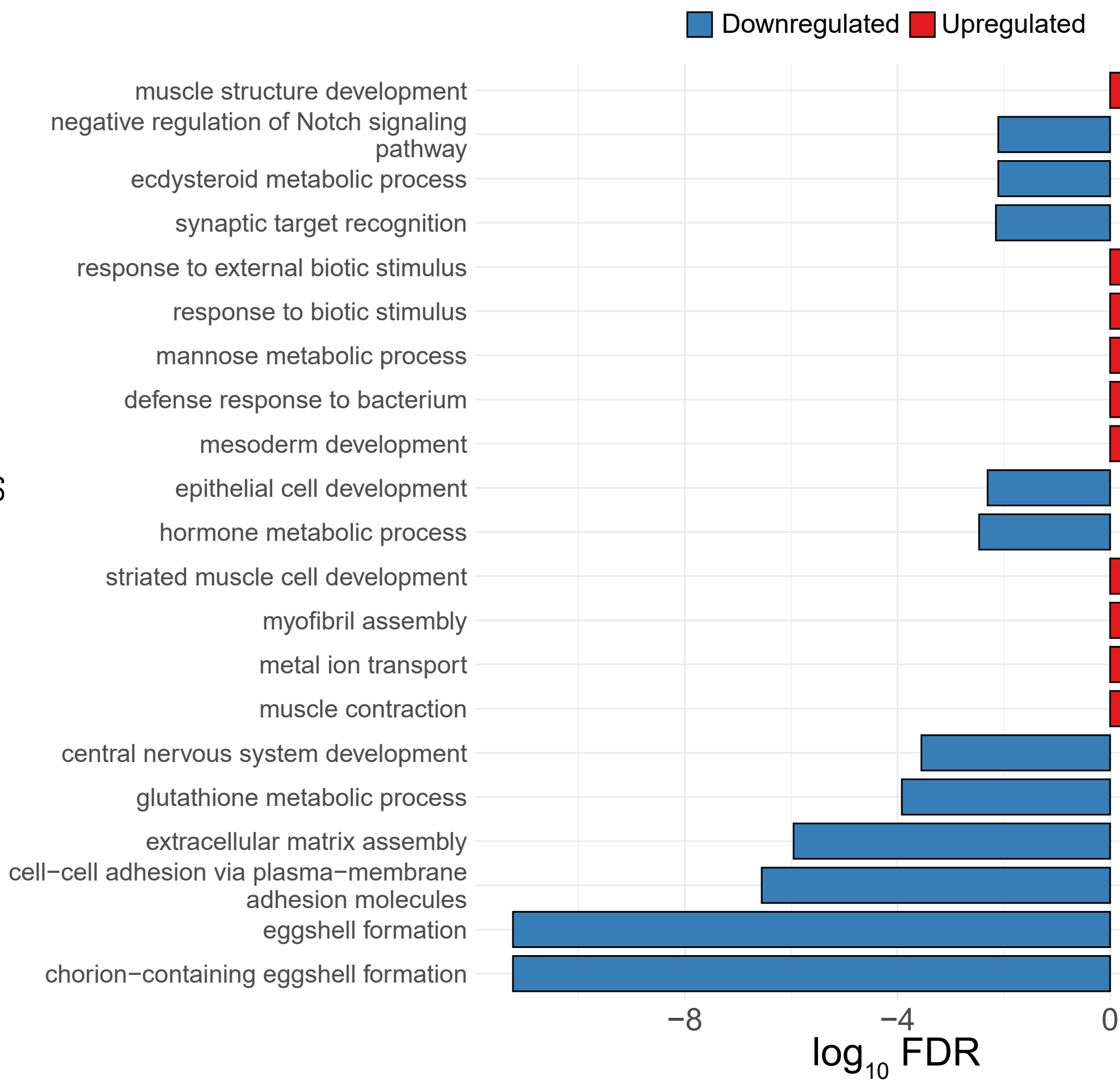**B**

Ontology

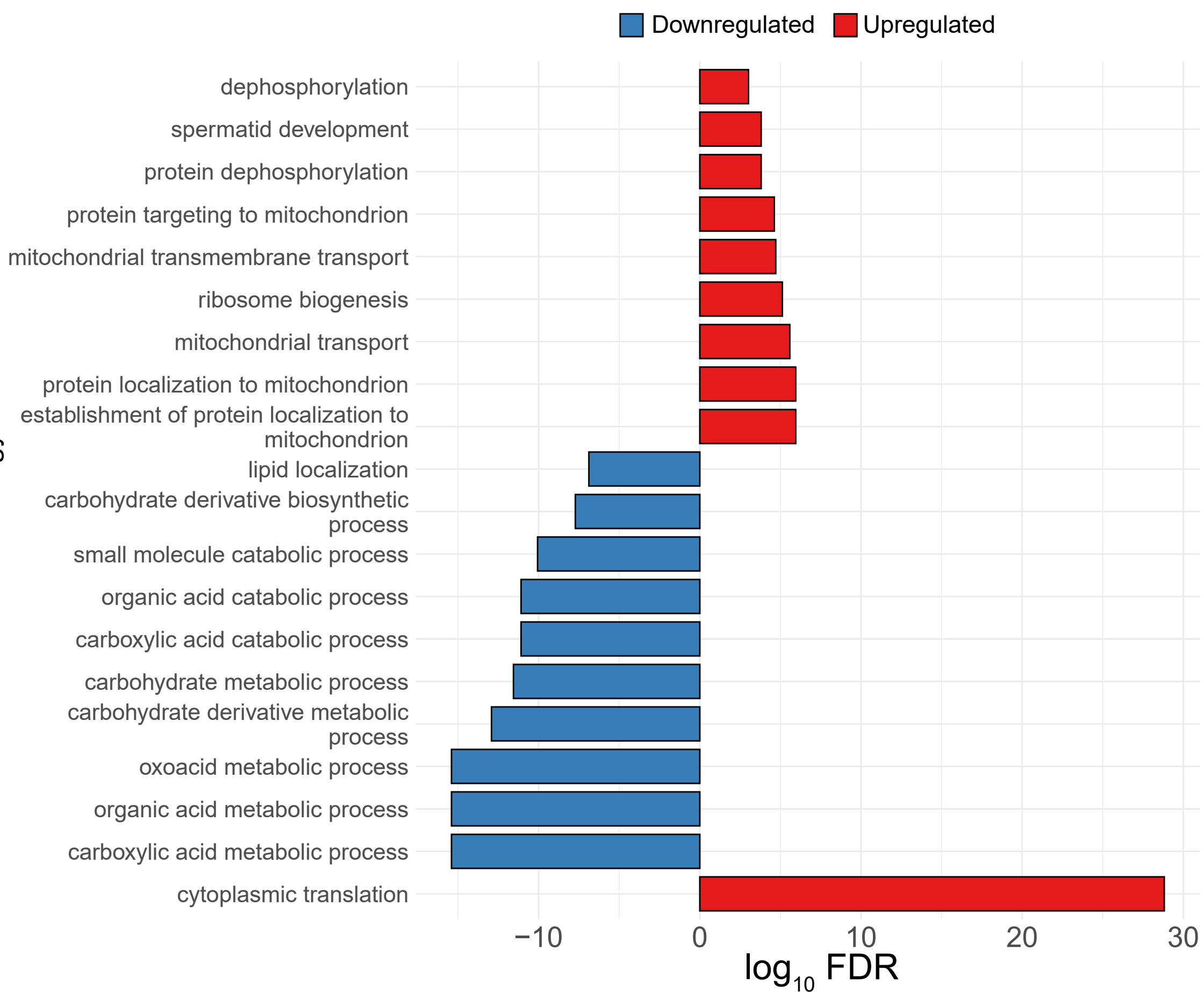

Supplement: Supplementary file 15 — Supplementary Material 15: Figure S4. GO enrichment analysis of the rapamycin main effects in the Abdomen. Biological processes enriched among DEGs upregulated (red) and downregulated (blue) by rapamycin in (A) Females, (B) Males. Top 10 upregulated and downregulated categories with the lowest FDR. All results in Supplementary Table S3. [file 12864_2024_10647_MOESM15_ESM.pdf]

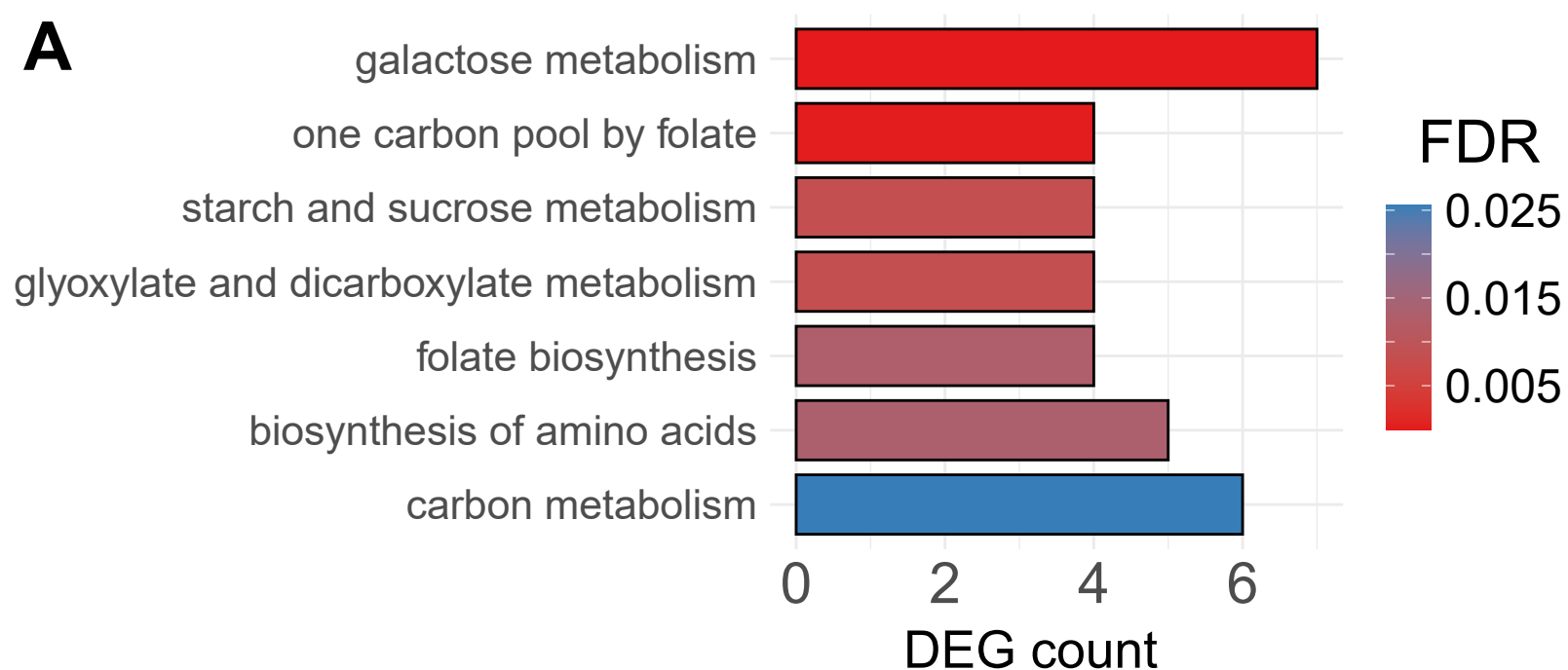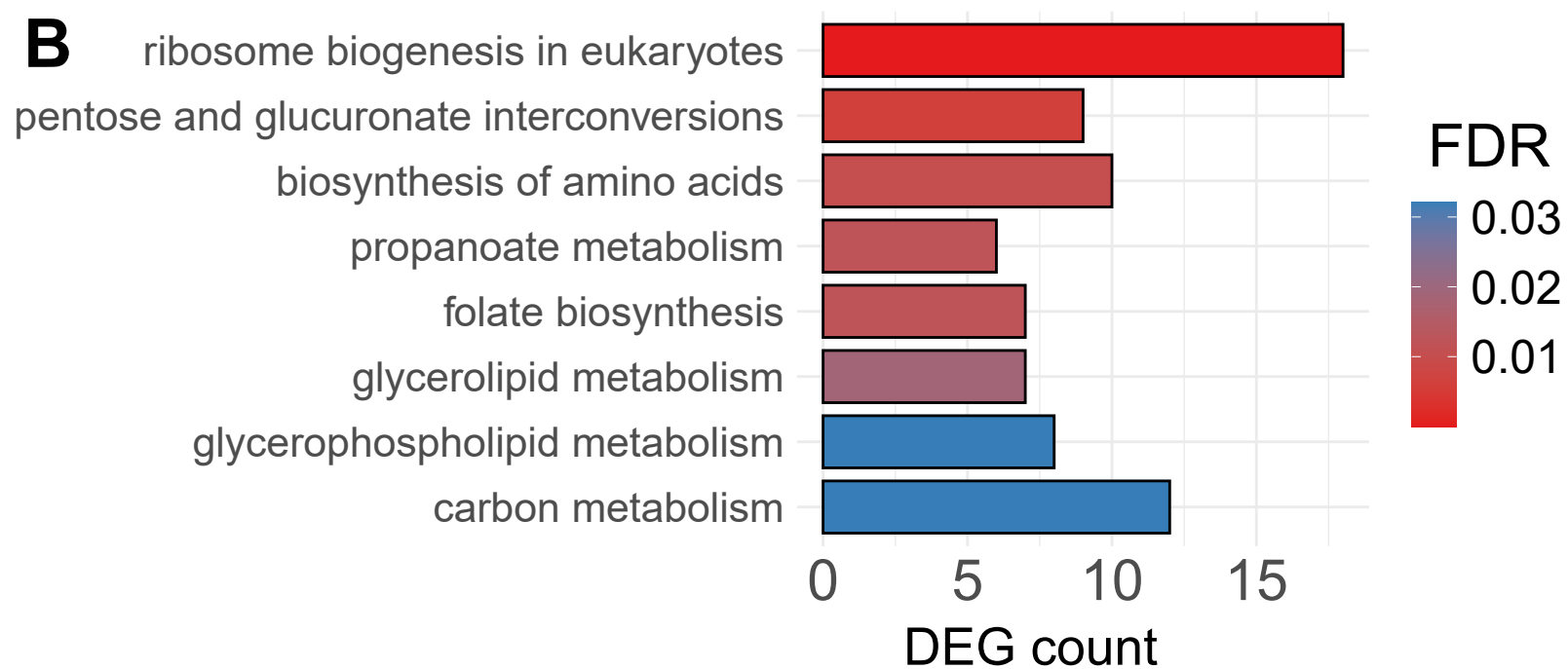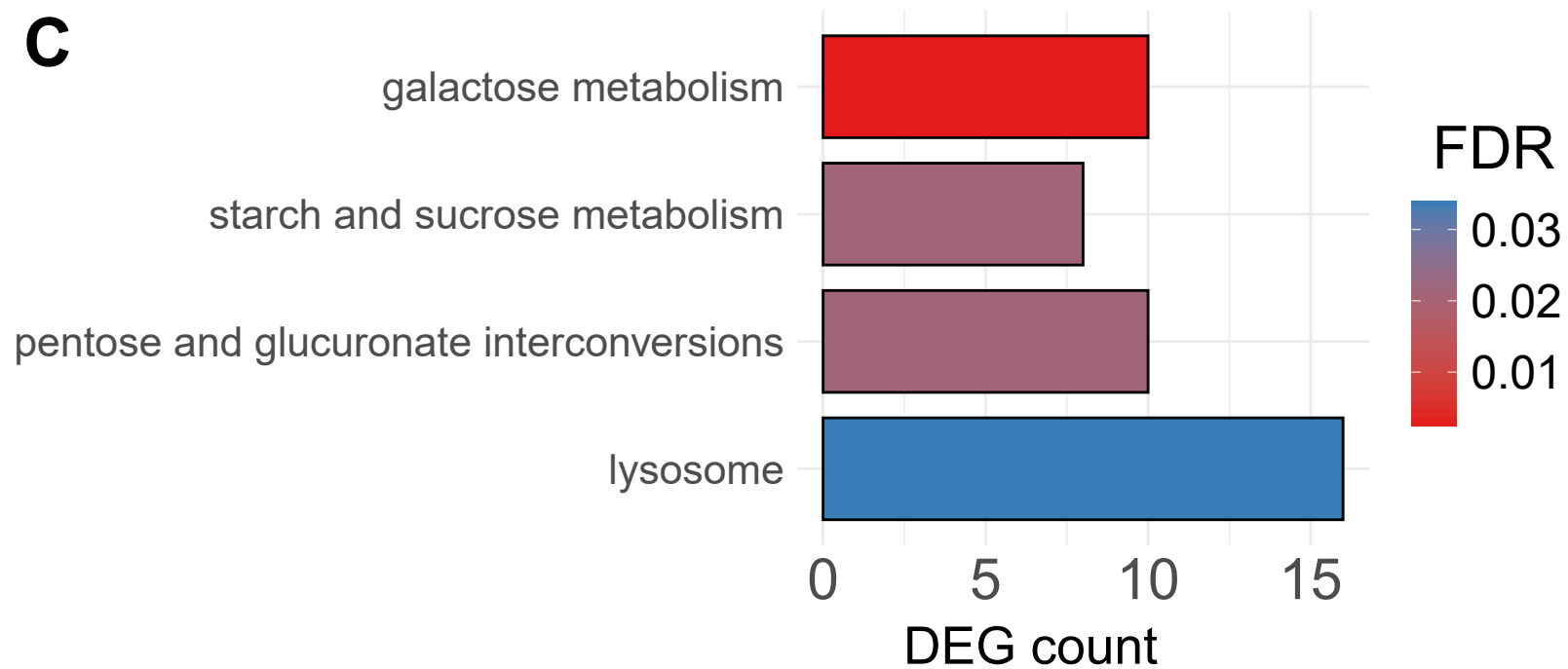

Supplement: Supplementary file 16 — Supplementary Material 16: Figure S5. KEGG enrichment analysis of the sex-by-treatment interaction. KEGG pathways enriched among the DEGs sensitive to sex-by-treatment interaction in (A) Thorax, (B) Head, and (C Abdomen. All results in Supplementary Table S5. [file 12864_2024_10647_MOESM16_ESM.pdf]

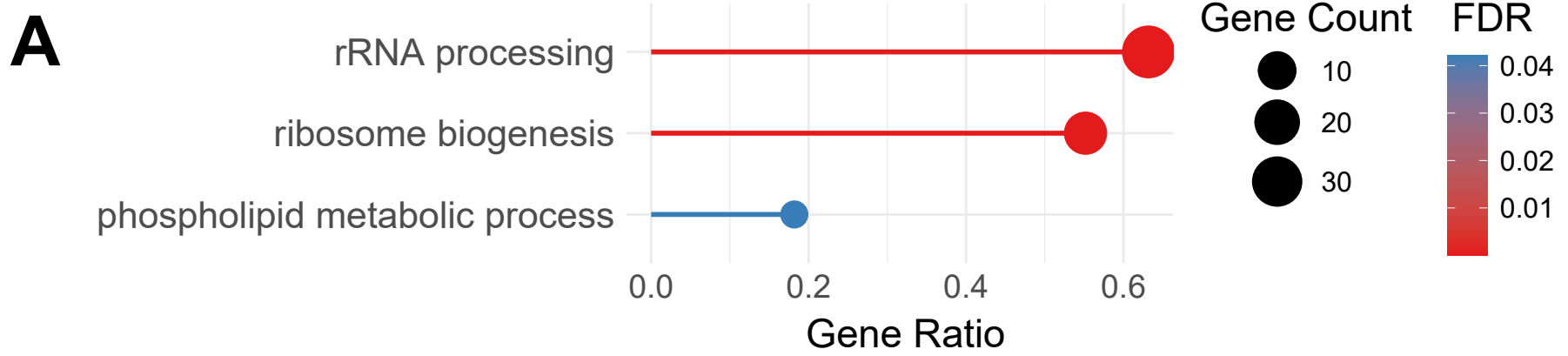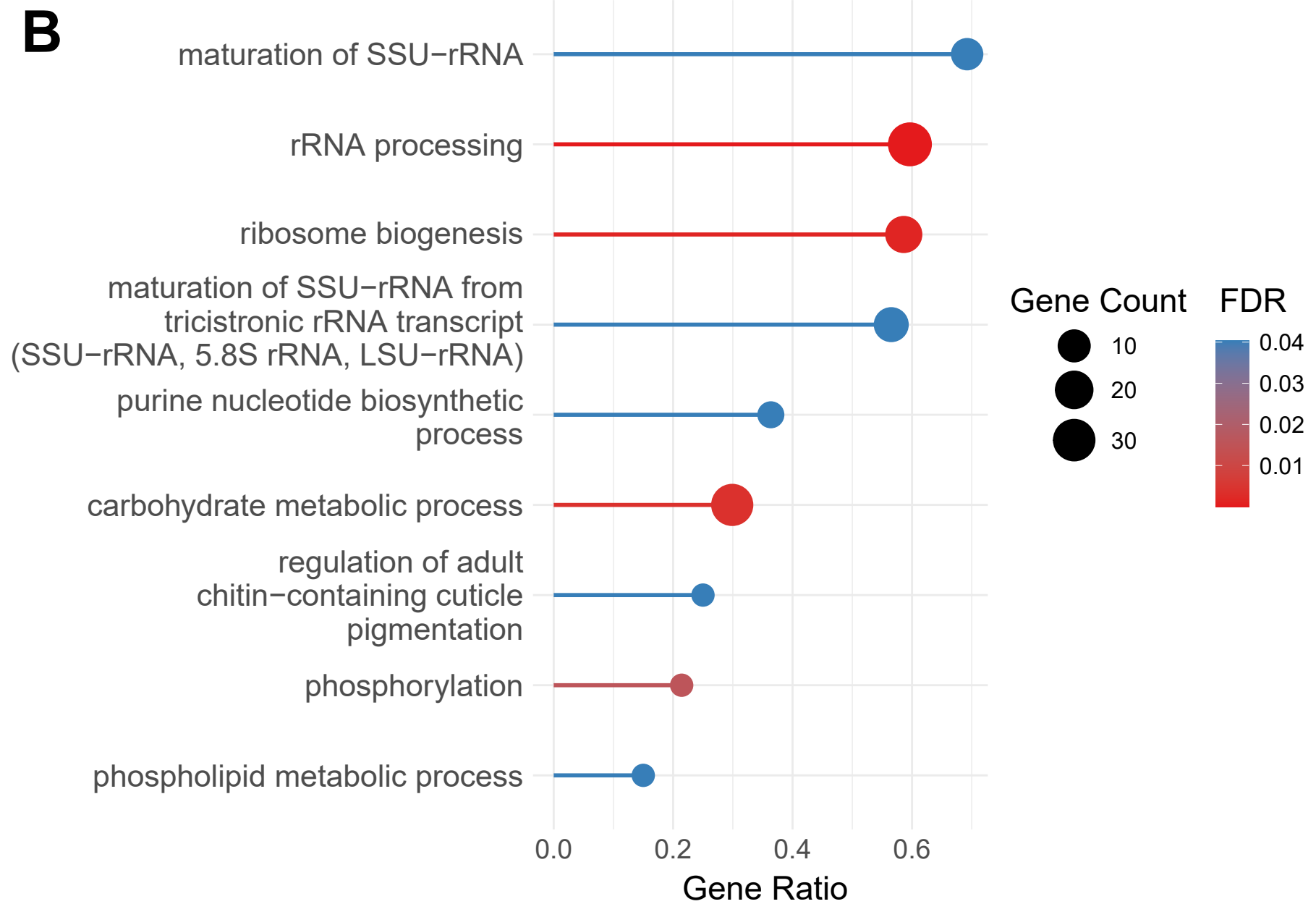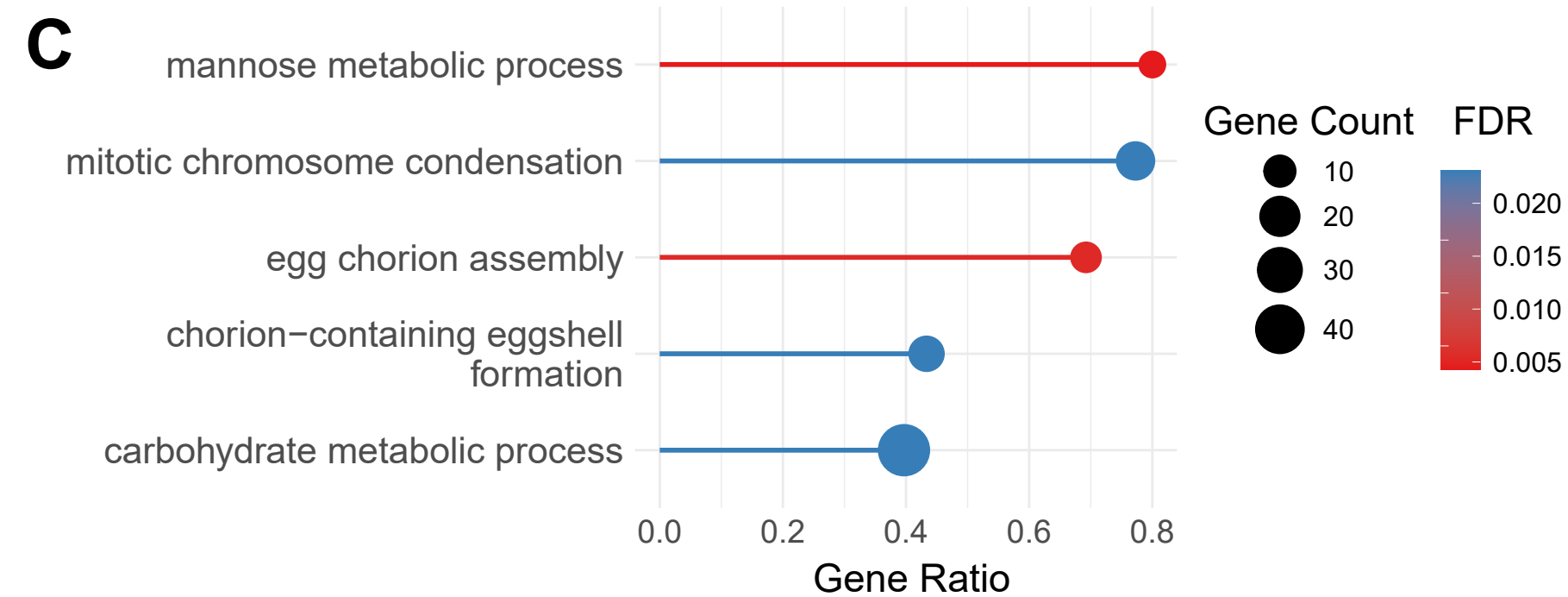

Supplement: Supplementary file 17 — Supplementary Material 17: Figure S6. Gene set enrichment analysis of the sex-by-treatment interaction. GSEA of GO biological processes among the DEGs sensitive to sex-treatment interaction in (A) Thorax, (B) Head, and (C) Abdomen. All results in Supplementary Table S5. [file 12864_2024_10647_MOESM17_ESM.pdf]

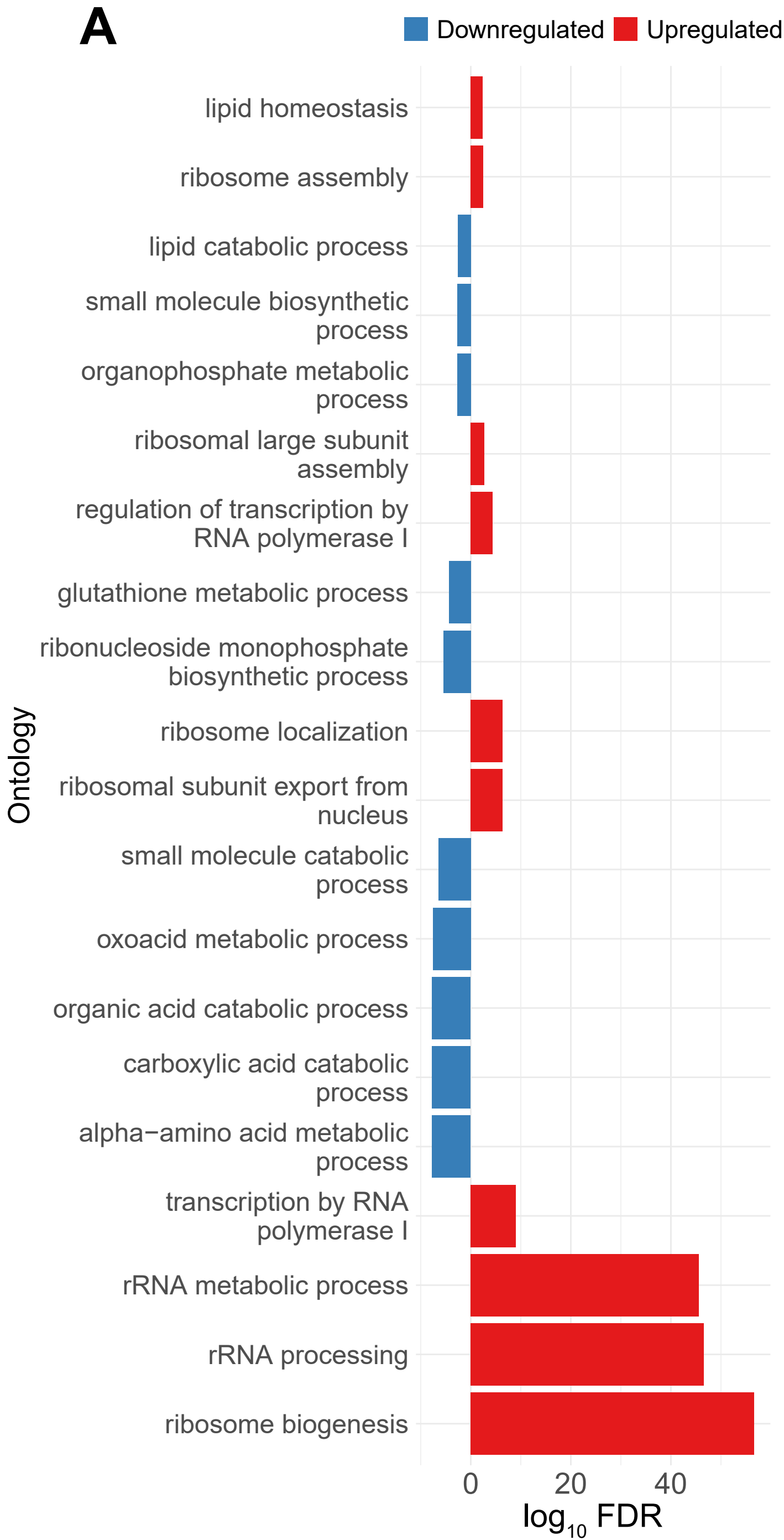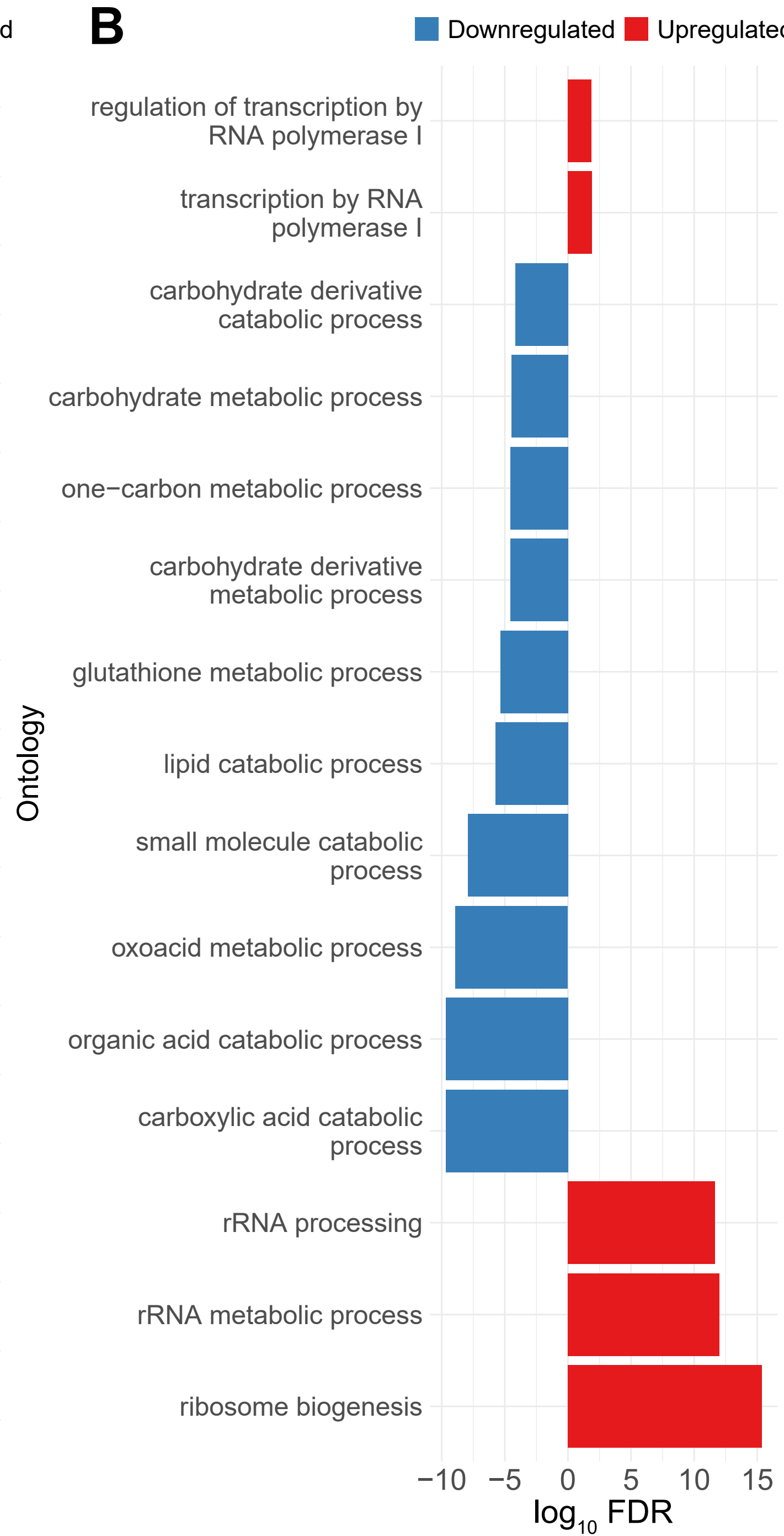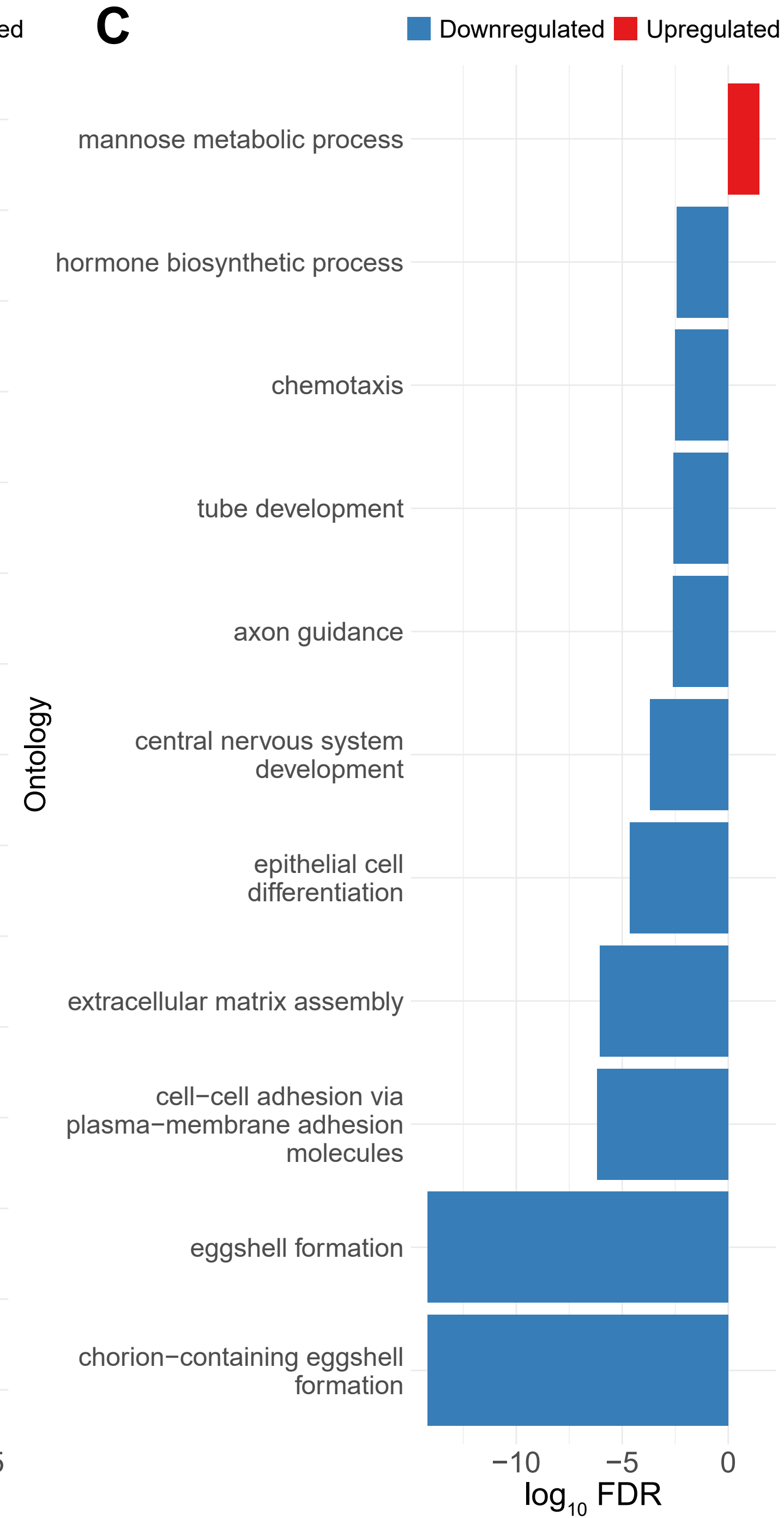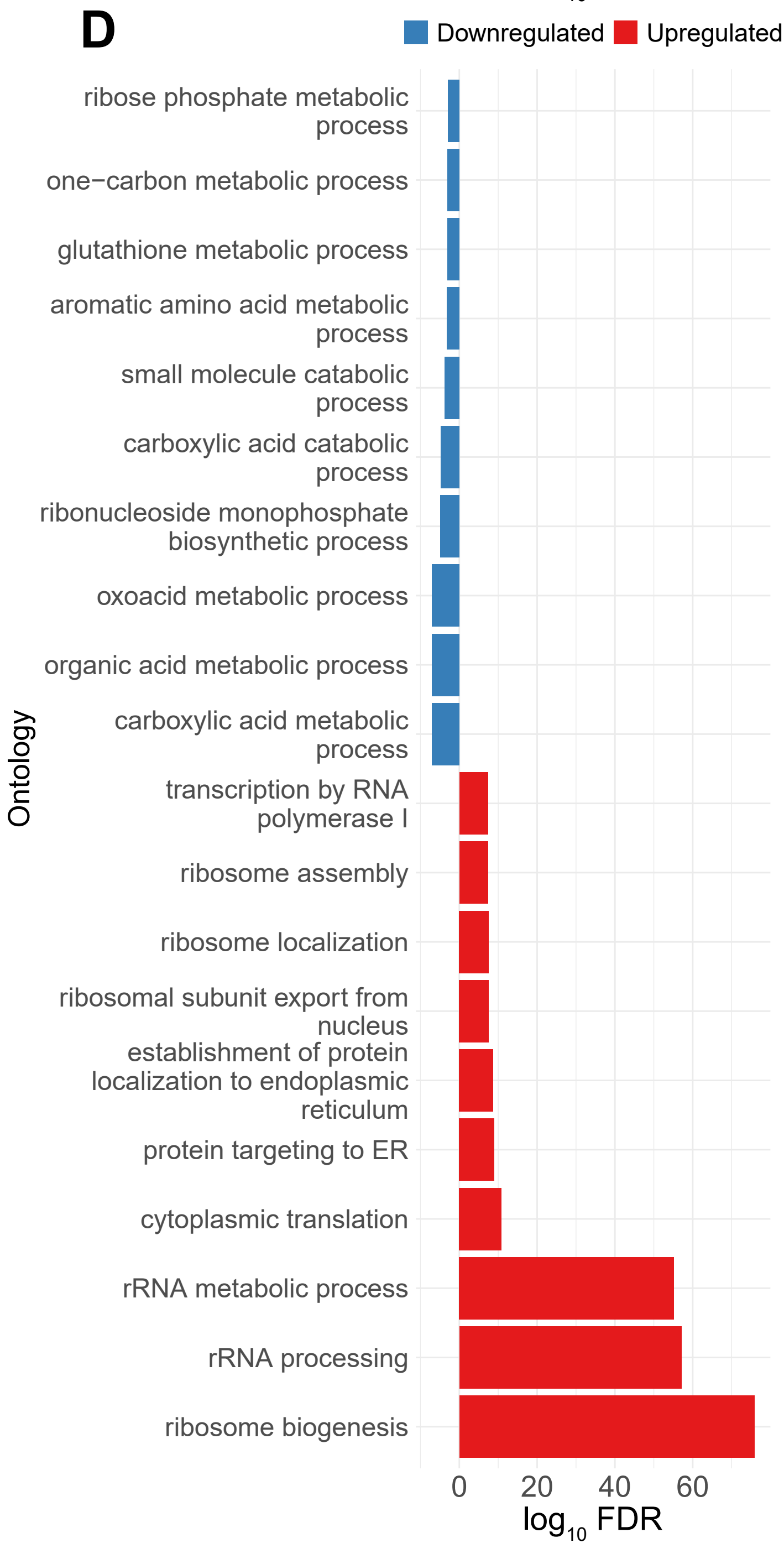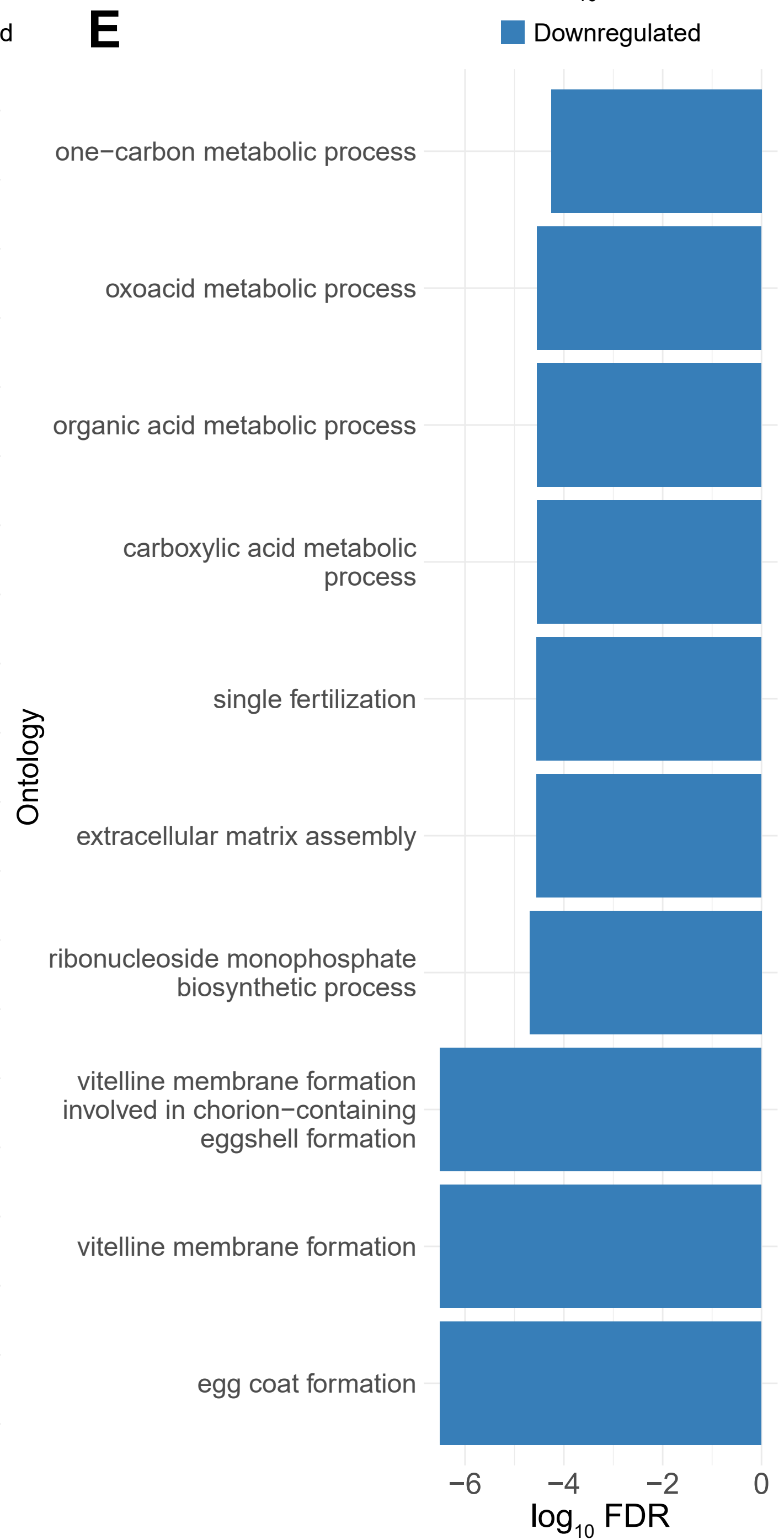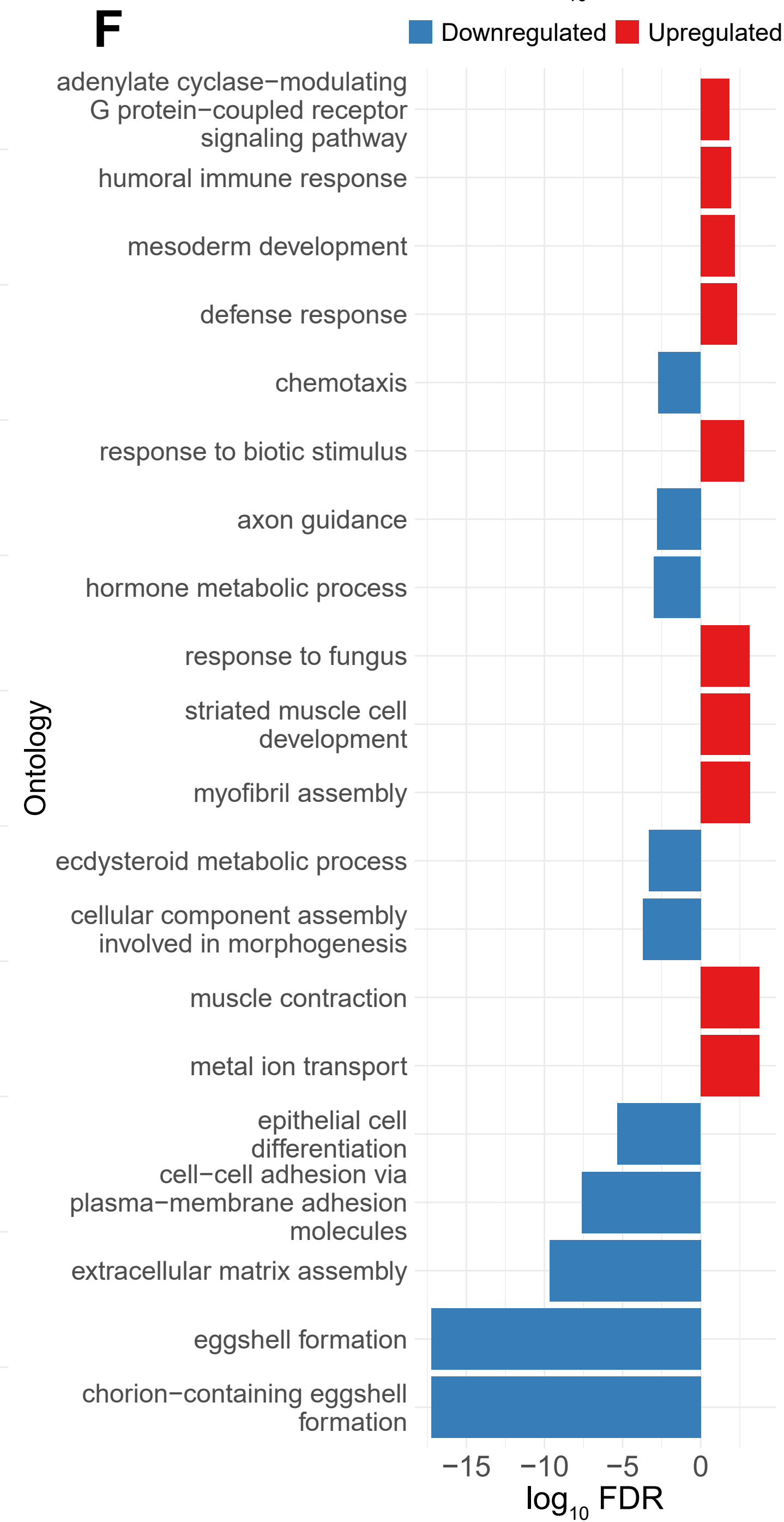

Supplement: Supplementary file 18 — Supplementary Material 18: Figure S7. GO enrichment analysis of the rapamycin main effects in female OreR;OreR and sm21;OreR flies. Biological processes enriched among DEGs upregulated (red) and downregulated (blue) by rapamycin in the head (A: OreR;OreR; D: sm21;OreR), thorax (B: OreR;OreR; E:sm21;OreR), and abdomen (C: OreR;OreR; F: sm21;OreR). Top 10 upregulated and downregulated categories with the lowest FDR. All results in Supplementary Table S9. [file 12864_2024_10647_MOESM18_ESM.pdf]

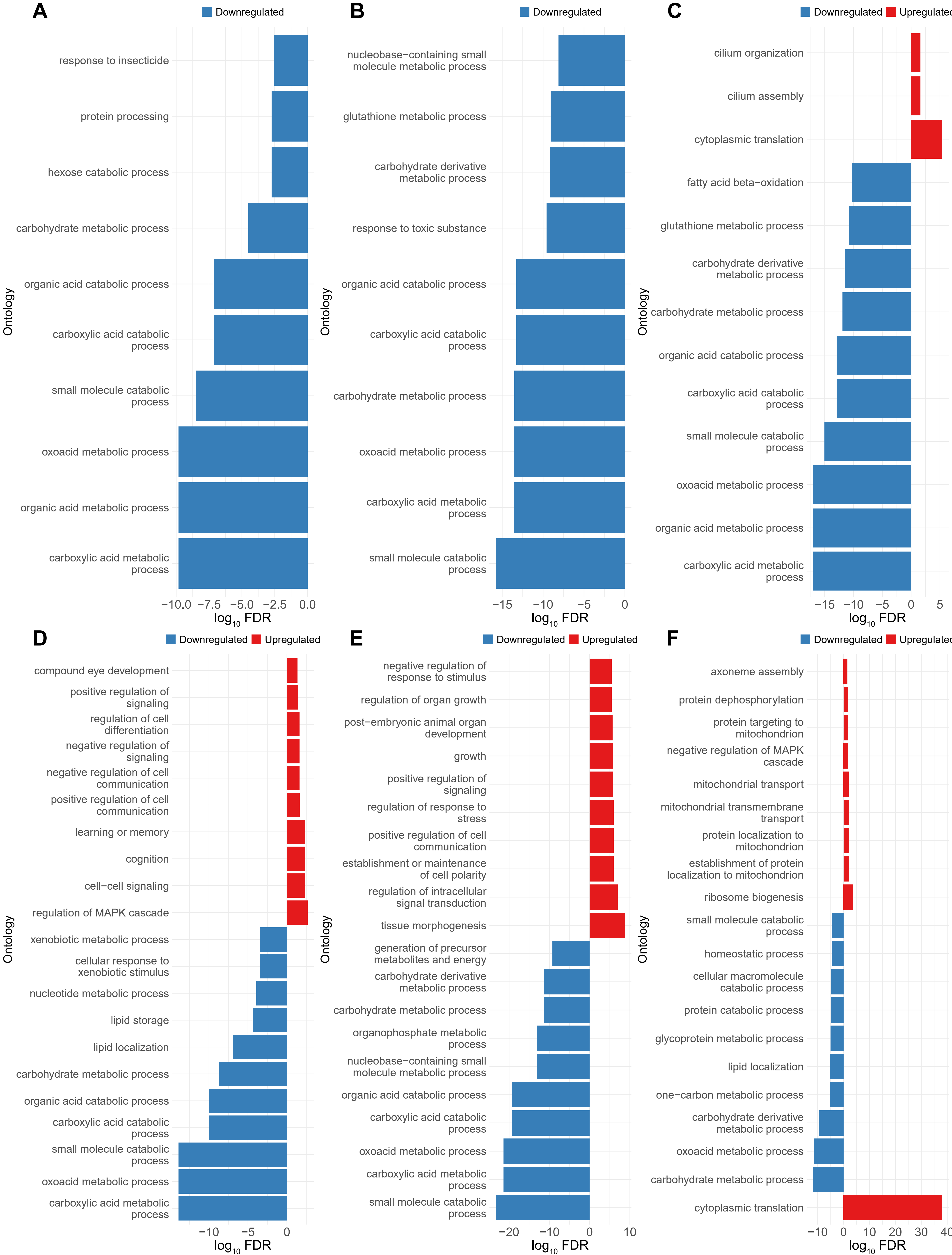

Supplement: Supplementary file 19 — Supplementary Material 19: Figure S8. GO enrichment analysis of the rapamycin main effects in male OreR;OreR and sm21;OreR flies. Biological processes enriched among DEGs upregulated (red) and downregulated (blue) by rapamycin in the head (A: OreR;OreR; D: sm21;OreR), thorax (B: OreR;OreR; E:sm21;OreR), and abdomen (C: OreR;OreR; F: sm21;OreR). Top 10 upregulated and downregulated categories with the lowest FDR. All results in Supplementary Table S9. [file 12864_2024_10647_MOESM19_ESM.pdf]
